# Long‐Term Cardiovascular Risk and Management of Patients Recorded in Primary Care With Unattributed Chest Pain: An Electronic Health Record Study

**DOI:** 10.1161/JAHA.121.023146

**Published:** 2022-03-18

**Authors:** Kelvin P. Jordan, Trishna Rathod‐Mistry, James Bailey, Ying Chen, Lorna Clarson, Spiros Denaxas, Richard A. Hayward, Harry Hemingway, Danielle A. van der Windt, Mamas A. Mamas

**Affiliations:** ^1^ School of Medicine Keele University Keele United Kingdom; ^2^ Department of Health and Environmental Sciences Xi'an Jiaotong–Liverpool University Suzhou China; ^3^ Institute of Health Informatics University College London London United Kingdom; ^4^ Health Data Research UK University College London London United Kingdom; ^5^ The National Institute for Health Research University College London Hospitals Biomedical Research Centre London United Kingdom; ^6^ Keele Cardiovascular Research Group School of Medicine Keele University Keele United Kingdom

**Keywords:** cardiovascular disease, chest pain, electronic health records, primary care, Epidemiology, Cardiovascular Disease

## Abstract

**Background:**

Most adults presenting with chest pain will not receive a diagnosis and be recorded with unattributed chest pain. The objective was to assess if they have increased risk of cardiovascular disease compared with those with noncoronary chest pain and determine whether investigations and interventions are targeted at those at highest risk.

**Methods and Results:**

We used records from general practices in England linked to hospitalization and mortality information. The study population included patients aged 18 years or over with a new record of chest pain with a noncoronary cause or unattributed between 2002 and 2018, and no cardiovascular disease recorded up to 6 months (diagnostic window) afterward. We compared risk of a future cardiovascular event by type of chest pain, adjusting for cardiovascular risk factors and alternative explanations for chest pain. We determined prevalence of cardiac diagnostic investigations and preventative medication during the diagnostic window in patients with estimated cardiovascular risk ≥10%. There were 375 240 patients with unattributed chest pain (245 329 noncoronary chest pain). There was an increased risk of cardiovascular events for patients with unattributed chest pain, highest in the first year (hazard ratio, 1.25 [95% CI, 1.21–1.29]), persistent up to 10 years. Patients with unattributed chest pain had consistently increased risk of myocardial infarction over time but no increased risk of stroke. Thirty percent of patients at higher risk were prescribed lipid‐lowering medication.

**Conclusions:**

Patients presenting to primary care with unattributed chest pain are at increased risk of cardiovascular events. Primary prevention to reduce cardiovascular events appears suboptimal in those at higher risk.

**Clinical Perspective**

**What Is New?**

- This study of over half a million patients using linked primary and secondary care data has shown that patients presenting to primary care and recorded with unattributed chest pain are at increased risk of coronary events over the following 10 years.
- Primary prevention, such as prescribing of lipid‐lowering medication, to reduce future cardiovascular events in those at higher risk appears suboptimal.

**What Are the Clinical Implications?**

- Better targeting to identify those most at risk for investigation and preventative measures may help reduce the population burden of cardiovascular events given the high incidence of patients with unattributed chest pain in primary care.

Each year, between 1% and 3% of adults will present for the first time in primary care with chest pain.^1^, ^2^, ^3^, ^4^, ^5^, ^6^ General practitioners or family physicians may pursue investigations in those for whom coronary heart disease is considered a diagnostic possibility, and may diagnose angina or a noncoronary cause such as gastroesophageal disease, musculoskeletal disease, or anxiety.^7^ Many patients, however, will not receive a specific diagnosis, but will be considered to have an unattributed cause for their chest pain.^1^, ^2^, ^8^ Risk factors for future cardiovascular events are more prevalent in those who have unattributed chest pain compared with the general population,^8^ and they have an increased future incidence of cardiovascular disease compared with those without chest pain.^9^, ^10^

In our previous study of 172 000 patients presenting for the first time with chest pain in UK primary care between 2002 and 2009,^4^ we reported that most patients did not have a diagnosis recorded at first presentation or in the next 6 months and generally did not undergo diagnostic testing. We found that those recorded with unattributed chest pain had an increased risk of cardiovascular events, and myocardial infarction specifically, over 5 years compared with those diagnosed with noncoronary chest pain. Although the risk of future myocardial infarction was lower than in patients initially diagnosed with angina, the absolute number of patients with a future myocardial infarction was nearly 5 times higher in the cohort with unattributed chest pain because of its greater size.^4^ Since the time period used in our previous study (2002–2009), there have been changes in the way that chest pain is investigated, with most high income countries implementing rapid‐access chest pain clinics in secondary and tertiary care for expeditious specialist review, investigation, and treatment, and adopting noninvasive investigations such as computed tomography coronary angiography and stress perfusion functional imaging, with more invasive investigations such as coronary angiography. The introduction of high‐sensitivity cardiac troponin assays able to detect smaller changes in cardiac enzymes released following myonecrosis has meant there has also been a change in the threshold for diagnosis of acute coronary syndromes/acute myocardial infarction, which may influence the relationships that we have reported previously.

The objectives of this study were first, to assess if there is an increased long‐term (10 year) risk of cardiovascular disease, coronary heart disease, myocardial infarction, and stroke in a national cohort of patients presenting to primary care with new unattributed chest pain compared with those recorded with a noncoronary cause of chest pain, and to assess how this risk varies over time. Second, we aimed to determine whether investigations and interventions in primary care are targeted at those most at risk and assess whether these have increased over time. We validated our findings in a second primary care electronic health records database.

## Methods

Data may be obtained from a third party and are not publicly available. The data were obtained from the Clinical Practice Research Datalink (CPRD). CPRD data governance does not allow us to distribute patient data to other parties. Researchers may apply for data access at http://www.CPRD.com/. Code lists used to define chest pain and cardiovascular disease are given in Table S1 and are also available from www.keele.ac.uk/mrr. The study was approved by the CPRD Independent Scientific Advisory Committee (ISAC ref 19_205) and the ISAC protocol made available to reviewers. CPRD has ethics approval from the Health Research Authority to support research using anonymized patient data. General practices provide consent for CPRD to collect de‐identified primary care data from their practice. Individual patients can opt‐out of sharing their data for research and CPRD does not collect data for these patients. Additional informed consent is not required. The data were analyzed in accordance with the relevant guidelines.

The primary analysis used the CPRD Aurum database, a primary care electronic health records database containing anonymized, routinely recorded information from over 20 million patients in over 1000 general practices in the United Kingdom that use EMIS Web software.^11^, ^12^ Practices used in this study were the subgroup of English practices (encompassing around 73% of patients at time of study) that have consented to linkage to inpatient diagnoses and procedures from Hospital Episode Statistics, cause‐specific mortality from the Office for National Statistics, and neighborhood deprivation scores.^13^

Validation of findings used the CPRD GOLD database,^12^, ^14^ which includes information from 17 million patients in over 800 general practices in the United Kingdom using Vision software, and was used for our previous study.^4^ Many general practices in England have switched from Vision to EMIS Web in recent years, hence many English practices whose records were in GOLD are now also included in Aurum, with patients lost to follow‐up in GOLD at point of migration. Given the size of Aurum, for this study, practices who changed software and hence whose records were included in both GOLD and Aurum were removed from the Aurum analysis data set to ensure sufficient numbers of practices in GOLD.

### Study Population

The study population was all patients aged 18 years or over with a first (incident) coded record of chest pain denoted as chest pain with cause unattributed, or chest pain attributed to a noncoronary cause, in primary care between 2002 and 2018. Those with a record of cardiovascular disease (as defined below) before their first recorded presentation of chest pain, or with <2 years of registration at their general practice at the time of their first chest pain event, were excluded. Index date was defined as the date of the first record of chest pain.

Up to 2018, UK primary care used Read codes to electronically record morbidity and processes of care. Our definition of unattributed chest pain included symptom codes not clearly specifying a cause of the pain, such as chest pain not otherwise specified and chest tightness. Noncoronary chest pain included recorded codes with specific attribution to organ systems other than cardiovascular such as costochondritis. Read code lists were derived through consensus work in our previous study.^4^

The first 6 months after index date was defined as the diagnostic window to allow time for investigations and diagnosis related to initial presentation to occur. We excluded those with a diagnosed cardiovascular disease in the first 6 months (diagnostic window) after index date and those with follow‐up time of <6 months from index date. Patients with initially unattributed chest pain at index date who received a record of noncoronary chest pain within the first 6 months were reallocated to the noncoronary group.

We also defined as a comparator group, patients with newly recorded angina, defined through a Read code or at least 2 prescriptions for nitrates. Those with <2 years prior registration or a recorded myocardial infarction in the first 6 months after index date were excluded.

### Outcomes

The primary outcome was a cardiovascular event defined as any of fatal or nonfatal acute myocardial infarction, angina, coronary heart disease not otherwise specified, heart failure, ventricular arrhythmia, cardiac arrest, ischemic stroke, hemorrhagic stroke, stroke type not specified, transient ischemic attack, peripheral arterial disease, abdominal aortic aneurysm, sudden cardiac death, and percutaneous coronary intervention and coronary artery bypass graft surgery.

Secondary outcomes were (1) coronary events including fatal or nonfatal acute myocardial infarction, angina, coronary heart disease not otherwise specified, percutaneous coronary intervention, coronary artery bypass graft surgery; (2) fatal or nonfatal acute myocardial infarction; and (3) stroke defined as ischemic stroke, hemorrhagic stroke, stroke type not specified, transient ischemic attack. Outcomes were captured from the primary and secondary care records and the Office for National Statistics death registry using previously derived and validated algorithms.^15^

Patients were followed from end of the 6‐month diagnostic window until earliest of outcome, end of their records in CPRD, and end of study period (December 31, 2018).

### Covariates

Covariates included demographic and risk factors for cardiovascular disease as included in the QRISK3 algorithm.^16^ QRISK3, recommended for use in UK primary care as a risk prediction tool in the general population, has been developed and validated on cardiovascular disease defined as coronary heart disease, ischemic stroke, and transient ischemic attack. We also included potential alternative explanations for chest pain and comorbidities identified previously as predictive of cardiovascular disease.^17^, ^18^ Demographic variables included age at index date, sex, race (defined as White or not recorded, and other ethnic groups), and neighborhood deprivation (based on Townsend score). Risk factors included smoking status, type 1 and type 2 diabetes, family history of coronary heart disease under age 60 years, chronic kidney disease, atrial fibrillation, treated hypertension, migraine, rheumatoid arthritis, severe mental illness (including schizophrenia, psychoses, bipolar disorder, and moderate/severe depression), corticosteroids prescription, body mass index, and total cholesterol to high‐density lipoprotein (HDL) ratio. Alternative explanations for chest pain and comorbidities considered predictive of cardiovascular disease included anxiety and mild depression, esophageal reflux, respiratory conditions (chronic obstructive pulmonary disease, chest infection, asthma), osteoarthritis, low back pain, neck pain, and cancer. Comorbidities were measured from 24 months before index date until end of the 6‐month diagnostic window. Prescription‐based covariates (treated hypertension, corticosteroids) were defined as at least 2 prescriptions of the relevant medication in this 30‐month time period. Total cholesterol to HDL ratio measurement, body mass index, and smoking status were based on record nearest, but before, the end of the 6‐month diagnostic window. Body mass index was categorized as normal or underweight, overweight, obese, or missing. Smoking was categorized as current, ex, never, or missing.

### Management

We determined the prevalence of cardiac diagnostic investigations and interventions (lipid‐lowering, antiplatelet, antidiabetes, antihypertensive prescriptions) during the 6‐month diagnostic window in those patients with unattributed chest pain and rated as elevated risk using the QRISK3 algorithm, developed and validated in UK national primary care electronic health records.^16^ Investigations included coronary angiography and computed tomography coronary angiography, as well as functional imaging including magnetic resonance imaging, echocardiography (stress, exercise), electrocardiogram (stress, exercise), and myocardial perfusion scans. The QRISK3 estimated risks were calculated using the online open access algorithm,^19^ replicated for use in Stata/MP 15.1 for Windows (StataCorp, College Station, TX), and compared for different combinations of risk factors to the estimated risk produced by the online calculator. A cutoff of 10% or more on the QRISK3 algorithm was used to indicate increased 10‐year risk of future cardiovascular disease as the recognized level of introducing preventative treatment.^20^ Although developed and validated in the general population, and therefore untested as an individual risk prediction model in this population, use of the QRISK3 algorithm here allows assessment of management in those patients likely to be at higher risk of cardiovascular disease. The algorithm was applied at the index date using the relevant covariates.

### Statistical Analysis

Incidence of cardiovascular and coronary events, myocardial infarction, and stroke were derived by type of chest pain per 10 000 person‐years. Flexible parametric survival analyses were used to compare risk of a long‐term outcome by type of chest pain, presented as hazard ratio (HR) with 95% CI. The optimal number of knots with evenly spaced centile positions for the restricted cubic splines were selected graphically and based on goodness‐of‐fit statistics, namely the Akaike Information Criterion and Bayesian Information Criterion. Models were derived unadjusted, adjusted for demographic characteristics and year of index date, further adjusted for factors included in QRISK3, and in the final model for all covariates. Because total cholesterol to HDL ratio was only recorded in 47% of patients in the chest pain groups, this covariate was included only in a sensitivity analysis by adding it to the final model and categorized as ≤4, 4 to 6, >6, and missing. A further sensitivity analysis added prescription of a lipid‐lowering medication to the final model. Time‐dependent effects were plotted over calendar time, with HRs computed at 12, 36, 60, and 120 months of follow‐up. Finally, interactions with year of index date were included to assess if risk estimates changed over calendar time, comparing index years 2002, 2006, 2010, and 2014 with follow‐up restricted to 36 months.

Robust standard errors to account for clustering in practices were used. Nonlinearity of relationships with outcome for age and body mass index were assessed using fractional polynomials, but did not improve model fit or alter findings, so only linear terms were retained. Death from noncardiovascular cause was included as a competing risk. However, because this did not change the estimated HRs, estimates from models without competing risks are presented.

Analyses were repeated for the secondary outcomes. Because angina is included in the definition of cardiovascular and coronary events, the angina group was only compared with the unattributed and noncoronary chest pain groups in relation to the myocardial infarction and stroke outcomes.

We also descriptively explored the extent that investigations and preventative medication had been used in the unattributed chest pain group, whether this has changed over time, and how this compares to use in the noncoronary chest pain group. The associations of the covariates with an investigation and with a new lipid‐lowering prescription (in those without such a prescription in the 24 months before index date) were assessed using binary logistic regression.

All analyses were conducted in CPRD Aurum and replicated in CPRD GOLD.

## Results

In CPRD Aurum, the population aged ≥18 years ranged from 3 603 905 in 2002 to 4 517 075 in 2018. There were 375 240 patients with newly recorded unattributed chest pain, 245 329 patients with noncoronary chest pain, and 24 554 patients with angina in CPRD Aurum between 2002 and 2018. Baseline characteristics are shown in Table 1. Patients with unattributed chest pain were older than those with noncoronary chest pain (mean age, 47.8 versus 45.5 years, respectively), but younger than those diagnosed with angina (mean, 66.3 years). The prevalence of comorbidities such as diabetes, hypertension, and atrial fibrillation were slightly higher in the unattributed chest pain compared with the noncoronary chest pain group but generally lower than in the group with angina. Median length of follow‐up was similar between the 2 chest pain groups (2244 days versus 2168 days) and slightly longer in the angina group (2625 days).

**Table 1:** Patient Characteristics by Type of Chest Pain in Aurum

	Chest pain noncoronary	Chest pain unattributed	Angina
N	245 329	375 240	24 554
Age, y, mean (SD)	45.5 (16.76)	47.8 (16.52)	66.3 (12.16)
Women	142 297 (58)	199 768 (53)	11 726 (48)
Race, White	203 447 (88)	311 523 (88)	22 415 (93)
Deprivation			
Least	50 462 (21)	81 264 (22)	5659 (23)
2nd	46 864 (19)	75 267 (20)	5065 (21)
3rd	47 532 (19)	71 354 (19)	4775 (19)
4th	46 129 (19)	67 448 (18)	4481 (18)
Most	54 137 (22)	79 587 (21)	4548 (19)
Risk factors			
Smoking			
Current	76 225 (32)	105 817 (29)	4895 (20)
Ex	51 018 (21)	85 705 (23)	8874 (37)
Never	110 437 (46)	173 272 (47)	10 125 (42)
Diabetes			
Type 1	949 (<1)	1347 (<1)	134 (<1)
Type 2	11 011 (4)	20 454 (5)	3986 (16)
FH, angina/heart attack age <60 y	8582 (3)	19 090 (5)	1970 (8)
Chronic kidney disease stage 3–5	10 505 (4)	19 298 (5)	3624 (15)
Atrial fibrillation	1734 (<1)	4774 (1)	1455 (6)
Treated hypertension	39 809 (16)	77 707 (21)	18 557 (76)
Migraine	7839 (3)	11 189 (3)	354 (1)
Rheumatoid arthritis	1592 (<1)	2366 (<1)	225 (<1)
Severe mental illness	4235 (2)	6974 (2)	389 (2)
Corticosteroid medication	12 855 (5)	20 170 (5)	2290 (9)
BMI, mean (SD)	26.5 (5.82)	26.9 (5.83)	28.6 (5.70)
Cholesterol/HDL ratio, mean (SD)	3.8 (1.36)	3.9 (1.44)	3.8 (1.42)
Alternative explanation/comorbidity			
Depression/anxiety	39 675 (16)	61 691 (16)	2701 (11)
Esophageal reflux	19 065 (8)	35 125 (9)	2330 (9)
Respiratory	50 768 (21)	78 811 (21)	5735 (23)
Osteoarthritis	11 374 (5)	18 438 (5)	2418 (10)
Low back pain	43 473 (18)	64 769 (17)	3244 (13)
Neck pain	17 622 (7)	25 873 (7)	1501 (6)
Cancer	5937 (2)	9529 (3)	927 (4)
QRISK3, median (IQR)	2.56 (0.53–9.19)	3.82 (0.88–11.79)	N/A

The values are presented as number (percent) unless stated otherwise. Complete data range: race 94% to 98%, smoking 97% to 97%, BMI 85% to 90%, total cholesterol to HDL ratio 38% to 77%. BMI indicates body mass index; FH, family history; HDL, high‐density lipoprotein; IQR, interquartile range; and QRISK3, score from the QRISK3 cardiovascular risk calculator.

In CPRD GOLD, there was a higher ratio of patients with unattributed chest pain (n=226 186) to noncoronary chest pain (n=89 145), but baseline characteristics for each chest pain group were similar to the corresponding group of patients in Aurum (Table S2). The median length of follow‐up was shorter in CPRD GOLD, with a median follow‐up of 1960 days for the unattributed chest pain group and 18% with 10 years of follow‐up compared with 27% in Aurum.

**Figure 1:**
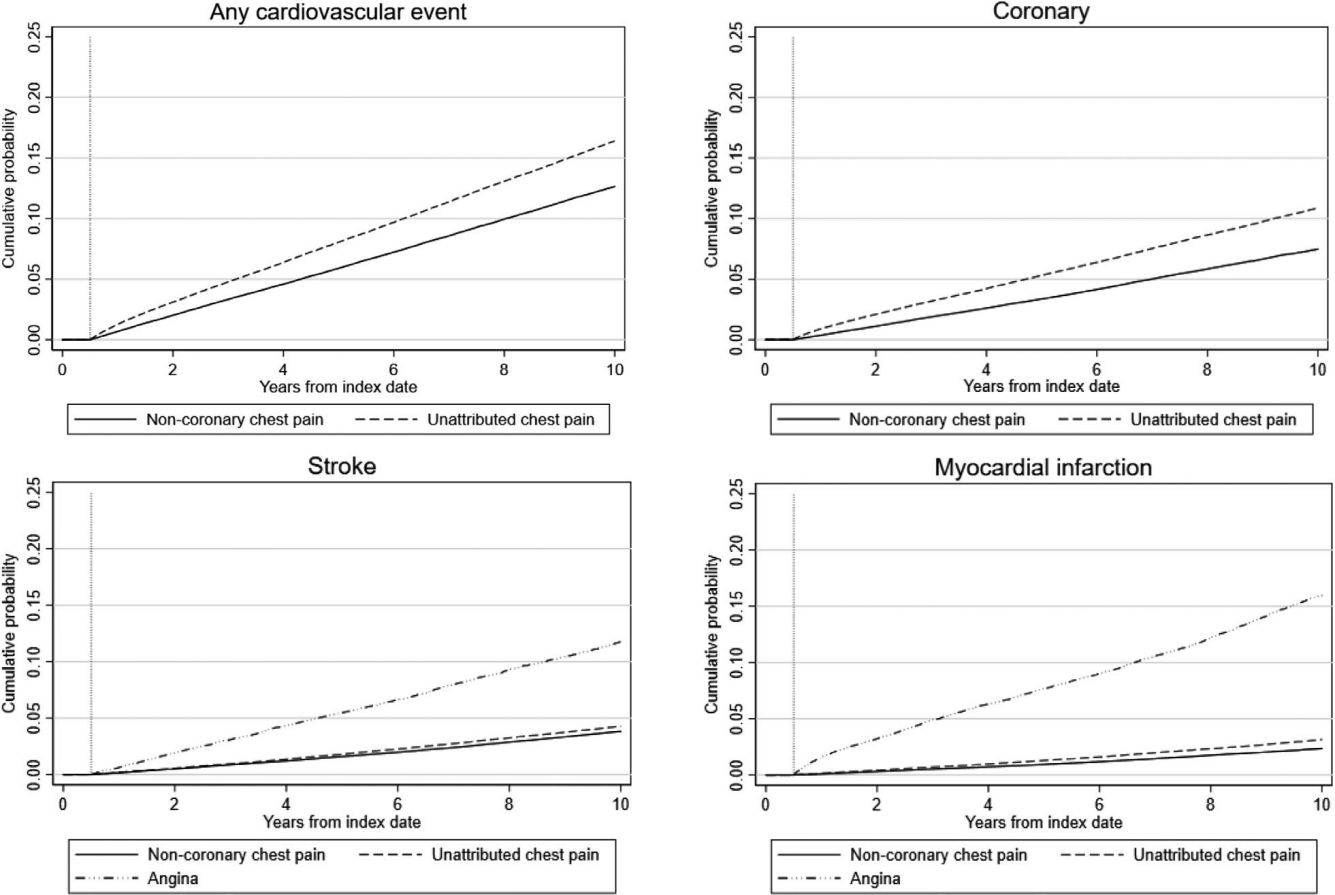
Kaplan‐Meier curves of time to first cardiovascular event from the end of the 6‐month diagnosis window in Aurum.

Figure 1 shows the Kaplan‐Meier curves for time to cardiovascular event using Aurum. During follow‐up, 11% (193/10 000 person‐years) of the unattributed chest pain group and 9% (144/10 000) of the noncoronary chest pain group had a recorded cardiovascular outcome. The fully adjusted flexible parametric model showed an overall increased risk of a cardiovascular event for patients with unattributed chest pain compared with noncoronary chest pain (HR, 1.16 [95% CI, 1.14–1.19; Table 2). Further adjustment for total cholesterol to HDL ratio and for lipid‐lowering prescription gave similar estimates (HR, 1.15 [95% CI, 1.13–1.17 and HR, 1.16 [95% CI, 1.13–1.18, respectively). Rates of cardiovascular events and the estimate of increased risk for patients with unattributed chest pain (HR, 1.13 [95% CI, 1.10–1.16) derived from GOLD were similar (Table 2). Table 3 and Figure S1 show the increased risk of a cardiovascular event for those with unattributed chest pain is highest in the first year after index date, but this risk remains even after 10 years (HR, 1.09 [95% CI, 1.06–1.13]). This increased risk for those with unattributed chest pain was consistent by calendar time (year of index date, Table S3).

**Table 2:** Incidence of Cardiovascular Events and Association With Unattributed Chest Pain Within Each Database

	No. at risk	With CVD, n (%)	Incidence of CVD per 10 000 person‐years	Unadjusted HR (95% CI)	Model 1 adjusted,* HR (95% CI)	Model 2 adjusted,^†^ HR (95% CI)	Model 3 adjusted,^‡^ HR (95% CI)
Aurum							
Noncoronary	245 329	21 195 (8.6)	144.30 (142.37–146.25)	1.00	1.00	1.00	1.00
Unattributed	375 240	42 982 (11.5)	193.02 (191.20–194.85)	1.34 (1.31–1.37)	1.19 (1.16–1.21)	1.16 (1.14–1.19)	1.16 (1.14–1.19)
GOLD							
Noncoronary	89 145	6666 (7.5)	143.93 (140.52–147.43)	1.00	1.00	1.00	1.00
Unattributed	226 186	21 508 (9.5)	184.48 (182.03–186.97)	1.28 (1.25–1.32)	1.16 (1.12–1.19)	1.13 (1.10–1.16)	1.13 (1.10–1.16)

CVD indicates cardiovascular disease; and HR, hazard ratio.

* Adjusted for age, sex, race, neighborhood deprivation, year of index presentation.

^†^ Additionally adjusted for smoking status, type 1 diabetes, type 2 diabetes, family history of coronary heart disease, chronic kidney disease, atrial fibrillation, treated hypertension, migraine, rheumatoid arthritis, severe mental illness, corticosteroid medication, body mass index.

^‡^ Additionally adjusted for depression/anxiety, esophageal reflux, respiratory, osteoarthritis, low back pain, neck pain, cancer.

**Table 3:** Association of Cardiovascular Events With Unattributed Chest Pain at Different Points During Follow‐Up in Aurum

	Time since index date			
	12 mo, HR* (95% CI)	36 mo, HR* (95% CI)	60 mo, HR* (95% CI)	120 mo, HR* (95% CI)
Any cardiovascular	1.25 (1.21–1.29)	1.11 (1.08–1.14)	1.09 (1.07–1.12)	1.09 (1.06–1.13)
Coronary	1.46 (1.41–1.52)	1.23 (1.19–1.27)	1.19 (1.15–1.22)	1.17 (1.13–1.22)
Myocardial infarction	1.15 (1.06–1.25)	1.17 (1.10–1.24)	1.18 (1.11–1.25)	1.12 (1.05–1.19)
Stroke	0.97 (0.91–1.03)	0.99 (0.95–1.04)	1.01 (0.96–1.06)	0.99 (0.94–1.04)

HR indicates hazard ratio.

* Fully adjusted model; noncoronary group is the reference group.

Patterns were similar when restricted to coronary outcomes (Figure 1, Tables 3 and 4) with those with unattributed chest pain having increased risk compared with the noncoronary chest pain group (HR, 1.30 [95% CI, 1.27–1.32]). This increased risk reduced but was still elevated over 10 years of follow‐up. Patients with unattributed chest pain also had increased risk of myocardial infarction (HR, 1.16 [95% CI, 1.12–1.20]). In contrast to any cardiovascular or coronary event, this increased risk estimate for myocardial infarction was consistent over the length of follow‐up but gradually increased over calendar time (index date in 2002: 3‐year HR, 1.11 [95% CI, 0.96–1.29]; index date in 2014: HR, 1.25 [95% CI, 1.12–1.39]; Table S3). There was no increased risk of a stroke in those with unattributed chest pain (HR, 0.99 [95% CI, 0.96–1.03]). Patients with angina had the highest risk of myocardial infarction (versus noncoronary HR, 2.50 [95% CI, 2.38–2.62]) and a smaller elevated risk of a stroke (HR, 1.11 [95% CI, 1.05–1.18]). These patterns and estimates were similar in the GOLD study population although with weaker associations at 10 years (Tables S4 and S5).

**Table 4:** Incidence of Types of Cardiovascular Events and Associations With Unattributed Chest Pain in Aurum

	No. at risk	With event, n (%)	Rate per 10 000 person‐years	Unadjusted, HR (95% CI)	Model 1, adjusted* HR (95% CI)	Model 2, adjusted^†^ HR (95% CI)	Model 3, adjusted^‡^ HR (95% CI)
Coronary							
Noncoronary	245 329	12 409 (5.1)	82.85 (81.41–84.32)	1.00	1.00	1.00	1.00
Unattributed	375 240	28 316 (7.6)	124.32 (122.88–125.78)	1.50 (1.47–1.53)	1.33 (1.30–1.36)	1.30 (1.27–1.33)	1.30 (1.27–1.32)
Myocardial infarction							
Noncoronary	245 329	3945 (1.6)	25.67 (24.88–26.48)	1.00	1.00	1.00	1.00
Unattributed	375 240	8193 (2.2)	34.46 (33.72–35.22)	1.34 (1.29–1.39)	1.16 (1.12–1.21)	1.16 (1.12–1.20)	1.16 (1.12–1.20)
Angina	24 554	3207 (13.1)	192.74 (186.18–199.52)	7.39 (7.06–7.74)	2.77 (2.64–2.91)	2.46 (2.34–2.58)	2.50 (2.38–2.62)
Stroke							
Noncoronary	245 329	6247 (2.6)	40.86 (39.86–41.88)	1.00	1.00	1.00	1.00
Unattributed	375 240	10 999 (2.9)	46.48 (45.62–47.36)	1.14 (1.10–1.18)	1.00 (0.97–1.04)	0.99 (0.96–1.03)	0.99 (0.96–1.03)
Angina	24 554	2292 (9.4)	135.98 (130.53–141.67)	3.28 (3.10–3.46)	1.19 (1.13–1.26)	1.08 (1.02–1.15)	1.11 (1.05–1.18)

HR indicates hazard ratio.

* Adjusted for age, sex, race, neighborhood deprivation, year of index presentation.

^†^ Additionally adjusted for smoking status, type 1 diabetes, type 2 diabetes, family history of coronary heart disease, chronic kidney disease, atrial fibrillation, treated hypertension, migraine, rheumatoid arthritis, severe mental illness, corticosteroid medication, body mass index.

^‡^ Additionally adjusted for depression/anxiety, esophageal reflux, respiratory, osteoarthritis, low back pain, neck pain, cancer.

**Figure 2:**
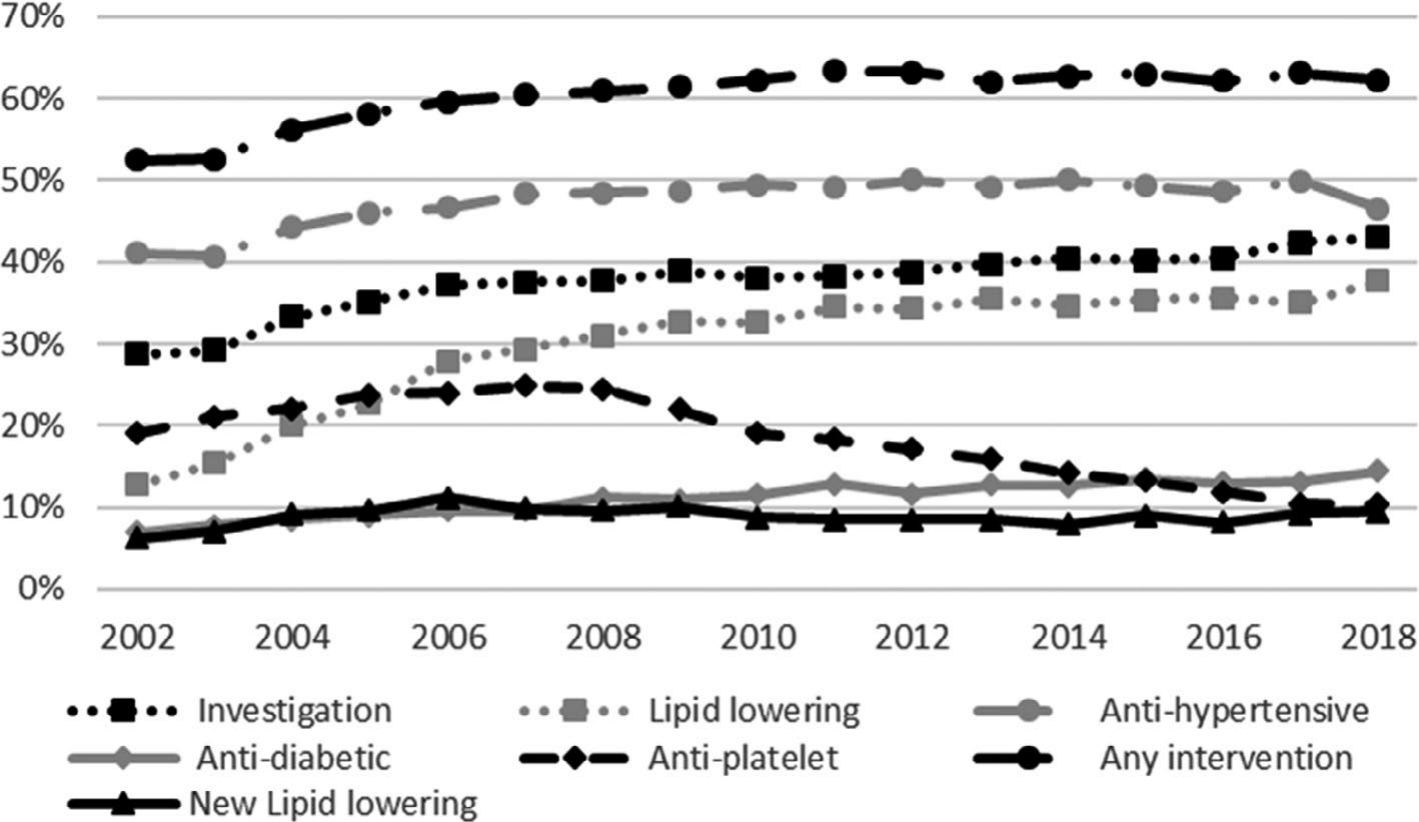
Investigations and interventions in the 6 months after first presentation with unattributed chest pain in those with risk ≥10% in Aurum. New lipid‐lowering drug denominator population excludes those with such a prescription in the 24 months before the index date.

Twenty‐nine percent of patients with unattributed chest pain and 23% with noncoronary chest pain had 10‐year risk scores of 10% or more. Thirty‐eight percent of the higher‐risk unattributed chest pain group had received an investigation in the 6 months after index consultation, and 60% were prescribed any of the 4 preventative and cardiovascular medications. Most (85%) of those receiving interventions were not newly prescribed this medication; they had a relevant recorded prescription medication in the 24 months before index date. Thirty percent were prescribed a lipid‐lowering drug during the 6 months after index consultation (Table 5). There was an increasing trend from 2002 to 2009 in investigations (Figure 2). There was variation in trends over time by intervention with prescribing of antiplatelets falling from 2008 and lipid‐lowering prescribing increasing up to 2011. However, the percentage of patients newly starting lipid‐lowering drugs has been stable at around 9% (Figure 2).

**Table 5:** Investigations and Interventions in the 6 Months After Index Date by Chest Pain Status in Aurum

	Noncoronary, n (%)		Unattributed, n (%)	
	All	10‐year QRISK3 ≥10%	All	10‐year QRISK3 ≥10%
Total	245 329	57 022	375 240	107 480
Investigation*	23 599 (10)	7403 (13)	113 297 (30)	40 411 (38)
Intervention				
Lipid lowering	19 318 (8)	14 901 (26)	43 620 (12)	32 121 (30)
Antihypertensive	37 702 (15)	25 425 (45)	76 761 (20)	51 147 (48)
Antidiabetes	8703 (4)	6540 (11)	15 233 (4)	11 910 (11)
Antiplatelet	8044 (3)	6554 (11)	27 573 (7)	19 951 (19)
Any intervention^†^	48 480 (20)	31 629 (55)	101 923 (27)	64 982 (60)
Neither	180 903 (74)	22 735 (40)	202 207 (54)	28 898 (27)
Investigation but no intervention	15 946 (6)	2658 (5)	71 110 (19)	13 600 (13)
Intervention but no investigation	40 827 (17)	26 884 (47)	59 736 (16)	38 171 (36)
Investigation and intervention	7653 (3)	4745 (8)	42 187 (11)	26 811 (25)
New lipid‐lowering intervention^‡^	2474 (1)	1599 (4)	11 691 (4)	7023 (9)

n=225 662 (noncoronary all), n=41 910 (noncoronary risk ≥10%), n=338 515 (unattributed all), n=79 276 (unattributed risk ≥10%). QRISK3 indicates score from the QRISK3 cardiovascular risk calculator.

* Coronary angiography and computed tomography coronary angiography, functional imaging including magnetic resonance imaging, echocardiography (stress, exercise), electrocardiogram (stress, exercise), and myocardial perfusion scans.

^†^ At least one prescription of lipid‐lowering, antihypertensive, antidiabetes, antiplatelet medication.

^‡^ In those with no prior lipid‐lowering prescription in 24 months before index date,

A new prescription of a lipid‐lowering drug was strongly associated with recorded total cholesterol/HDL ratio (odds ratio >6 versus ≤4, 2.68 [95% CI, 2.45–2.95]). Strong associations were also found with type 2 diabetes, family history of cardiovascular disease under age 60 years, age 55 to 74 years, and obesity (Table 6). Chronic kidney disease was associated with a reduced likelihood of such a prescription.

**Table 6:** Associations With Investigation or Intervention in Those With Unattributed Chest Pain and Cardiovascular Risk ≥10% at Baseline in Aurum

	Investigation, OR (95% CI)	New lipid‐lowering prescription,* OR (95% CI)
Age, y		
18–44	0.96 (0.88–1.06)	0.80 (0.67–0.96)
45–54	1	1
55–64	0.98 (0.94–1.03)	1.16 (1.07–1.26)
65–74	0.94 (0.89–0.98)	1.27 (1.16–1.38)
75+	0.81 (0.76–0.85)	0.89 (0.80–0.99)
Sex		
Men	1	1
Women	0.90 (0.88–0.93)	0.99 (0.93–1.05)
Race		
Other ethnic groups	1	1
White	1.19 (1.09–1.31)	1.06 (0.95–1.18)
Deprivation		
Least	1	1
2nd	0.95 (0.90–1.00)	0.97 (0.90–1.05)
3rd	0.90 (0.85–0.95)	0.99 (0.91–1.08)
4th	0.84 (0.79–0.90)	1.05 (0.96–1.15)
Most	0.69 (0.63–0.75)	1.04 (0.95–1.13)
Diabetes, type 2	0.85 (0.81–0.89)	1.82 (1.66–2.00)
FH		
Angina/heart attack age <60 y	1.45 (1.37–1.53)	1.74 (1.60–1.89)
Chronic kidney disease stage 3–5	0.98 (0.94–1.02)	0.85 (0.77–0.94)
Atrial fibrillation	1.51 (1.39–1.63)	1.13 (0.96–1.32)
Treated hypertension	0.92 (0.89–0.95)	1.05 (1.00–1.11)
Total/HDL ratio		
≤4	1	1
4–6	1.04 (1.01–1.08)	1.52 (1.44–1.62)
>6	1.02 (0.96–1.08)	2.68 (2.45–2.95)
Not recorded	0.55 (0.52–0.59)	0.32 (0.29–0.36)
BMI^†^		
Normal	1	1
Underweight	0.79 (0.69–0.89)	0.72 (0.54–0.96)
Overweight	1.10 (1.07–1.14)	1.20 (1.13–1.29)
Obese	1.14 (1.10–1.19)	1.29 (1.19–1.39)

BMI indicates body mass index; FH, family history; HDL, high‐density lipoprotein; and OR, odds ratio.

* In those with no lipid‐lowering prescription in 24 mo before index date, n=79 726.

^†^ Missing data means not recorded (not presented here).

## Discussion

This study of over half a million patients using linked primary and secondary care data, with findings validated in a second data set, has shown there is an increased long‐term risk of cardiovascular events in patients recorded in primary care with unattributed chest pain. We show that although over 1 in 4 patients appear at high risk of future cardiovascular events, only 38% of this group had an investigation and less than a third received a lipid‐lowering prescription.

Our study confirms that patients with unattributed chest pain have increased risk of future cardiovascular disease compared with those with noncoronary chest pain. Our previous study was restricted to a 5‐year follow‐up.^4^ Although the increased risk for any cardiovascular event is particularly elevated in the first 12 months after initially consulting primary care, importantly, our study indicates this risk is likely to continue over the long‐term (for at least 10 years). The increased risk of cardiovascular events appears less related to cerebrovascular events. This would suggest that this increased risk is not just related to an adverse cardiovascular risk factor profile and increased general risk of atherosclerotic events. It is possible some patients in the unattributed chest pain group may have undiagnosed markers of cardiovascular disease, although the prevalence of risk factors was similar (with the exception of essential hypertension and being slightly older) to the noncoronary chest pain group and was consistently lower than the angina group.

The high incidence of unattributed chest pain in primary care, and that 1 in 10 of these patients will develop a cardiovascular event over the next 10 years, suggests identifying those in this group most at risk and targeting them for investigation and preventative measures may be most beneficial in reducing the population burden of cardiovascular events.^8^ A greater targeted adoption of noninvasive investigations (for example, computed tomography coronary angiography^21^) in those with a high‐risk profile, may help to refine risk stratification in this population. Alongside prescribing of lipid‐lowering and other preventative medication, initiatives to increase physical exercise and change lifestyle behaviors such as improved diet and reduced smoking could help reduce future cardiovascular events in those at greatest risk.

In this study we used the QRISK3 algorithm to identify those who might be considered to be most at risk when they first present. QRISK3 was developed and validated for use in UK primary care to estimate the risk of cardiovascular events over 10 years in patients known to be currently free of cardiovascular disease and not currently prescribed lipid‐lowering medication. Hence, it needs validation and potential modification in the subgroups examined here. Although caution is needed in applying it to individual patients with unattributed chest pain, and QRISK3 may not be regularly used by clinicians, it contains the cardiovascular risk factors likely to be considered by a clinician when consulting with a patient with chest pain. Using QRISK3, we report that over 25% may have a high 10‐year risk of future cardiovascular events, higher than the 17% reported in the original QRISK3 general population validation cohort. Current guidelines suggest that patients at higher risk should be prescribed lipid‐lowering medications and aggressive primary prevention. Lipid‐lowering medications were prescribed in only 30% of patients with the higher‐risk cardiovascular disease profiles. The majority of those receiving lipid‐lowering medication in the 6 months after initial consultation for chest pain were already receiving these medications at time of initial chest pain consultation. Previous studies in the general population have suggested a discordance between cardiovascular risk as measured by algorithms and subsequent prescribing of lipid‐lowering medication.^22^, ^23^, ^24^ One UK primary care study between 2008 and 2010 showed 29% of patients previously not prescribed lipid‐lowering medication and who met the existing (2005) UK cardiovascular guidelines started lipid‐lowering medication over a 2‐year period.^22^ Similar to our study, they found increasing age, diabetes, total cholesterol level, family history of coronary heart disease, and prescription of antihypertensive drugs were all associated with new prescribing of lipid‐lowering medication. A general population study between 2007 and 2011 in primary care electronic health records identified the likelihood of a prescription of statins increased as 10‐year estimated cardiovascular risk increased, with around 28% of those with a QRISK2 score of ≥10% receiving a statin.^23^ This is similar to the level of recorded prescriptions based on the later QRISK3 algorithm found in this study for the same time period. A further study identified a 21% initiation of statins in patients aged 40 to 85 years with a QRISK2 score of ≥10% between 2012 and 2015, higher than our estimate of 9% initiation.^24^ We identified a reduction in use of antiplatelets since 2008, which may relate to uncertain evidence of its relative effectiveness for primary prevention in relation to adverse events.^25^

### Strengths and Limitations

A strength of this study was the large size of the cohort and long follow‐up, derived from an electronic health records database covering a nationally representative population drawn from across England,^11^, ^14^ with linkage of primary care information to inpatient hospital, mortality, and deprivation data. Similar data sets have been used previously to derive and validate the QRISK3 algorithm currently used in UK primary care to assess cardiovascular risk.^16^ A unique feature of this study was the opportunity to validate findings in a second national database, which suggests generalizability across England. The one difference between the findings in Aurum and GOLD was the weaker association between chest pain and cardiovascular events at 10 years. This may be because of the shorter length of follow‐up for patients included in GOLD.

The coded primary care record reflects the general practitioner’s opinion of the chest pain, including findings from any cardiac diagnostic investigation they have requested, on whether it is likely to be coronary. For the unattributed chest pain group, it does not indicate the general practitioner’s suspected underlying reason for the chest pain. This may be recorded in free (unstructured) text that generally cannot be accessed for research. It is possible angina was an underlying reason for some of the unattributed group, and undiagnosed angina has been shown to be as associated with poorer prognosis, including myocardial infarction and mortality, as those with diagnosed angina.^26^ The highest level of relative risk at 12 months after initial consultation may relate to there being an investigation and diagnostic period longer than 6 months for some patients before a cardiovascular event such as angina being diagnosed, perhaps because of symptomatic coronary heart disease with atypical presentation features. However, the introduction of rapid access chest pain clinics should ensure most patients receive a diagnosis within 6 months. Furthermore, there has been a reducing trend in both primary and secondary care recorded angina in recent years,^4^, ^27^ and the continued increased risk over 10 years and the consistent level of increased risk for myocardial infarction suggests unattributed chest pain is a long‐term risk factor for cardiovascular disease, and coronary events in particular. Other potential markers of cardiovascular risk such as coronary artery calcium score and echo parameters are not usually captured within primary care records and would not have been measured for most of our study population. A limitation is the level of missing data for some of the covariates; notably there were missing data on cholesterol level in around half of patients in both the noncoronary and unattributed chest pain groups. Given cholesterol is a strong risk factor within the cardiovascular risk algorithm, it is not unexpected that there were fewer missing data in those with cardiovascular risk scores over 10%. Overall, the mean total/HDL cholesterol ratios were similar between the 2 chest pain groups (3.8 versus 3.9), and our sensitivity analysis including categorized total/HDL cholesterol ratio as a covariate did not change our findings. As might be expected, new lipid‐lowering prescriptions were more likely to be issued to those with recorded high ratios, and less likely in those without a record of cholesterol level. It is likely that cholesterol tests were more common in those suspected of having high levels and to be at greater cardiovascular risk.

## Conclusions

Patients presenting to primary care and recorded with unattributed chest pain are at increased risk of coronary events over at least the following 10 years. However, current primary prevention to reduce future cardiovascular events appears suboptimal, even in those at higher risk. Better targeting to identify those most at risk for investigation and preventative measures may help reduce the population burden of cardiovascular events given the high incidence of patients with unattributed chest pain in primary care.

## Sources of Funding

The study was funded by the British Heart Foundation (ref PG/19/46/34307). K.P.J. is supported by matched funding awarded to the National Institute for Health Research Applied Research Collaboration (West Midlands). H.H. is a National Institute for Health Research Senior Investigator. His work is supported by Health Data Research UK (grant number LOND1), the National Institute for Health Research University College London Hospitals Biomedical Research Center, and the BigData@Heart Consortium, funded by the Innovative Medicines Initiative‐2 Joint Undertaking under grant agreement number 116074. The views and opinions expressed are those of the authors and not necessarily the views of the funders, National Health Service, National Institute for Health Research, or Department of Health and Social Care.

## Disclosures

None.

## Supporting information

Tables S1–S5
Figure S1
