# Supplementary material for: Long‐Term Cardiovascular Risk and Management of Patients Recorded in Primary Care With Unattributed Chest Pain: An Electronic Health Record Study
Source: J Am Heart Assoc. 2022 Mar 18;11(7):e023146. doi: 10.1161/JAHA.121.023146 (PMC9075433; doi:10.1161/JAHA.121.023146)
Supplement: Supplementary file 1 — Tables S1–S5 Figure S1 [file JAH3-11-e023146-s001.pdf]

SUPPLEMENTAL MATERIAL

Table S1 – Code lists for chest pain and cardiovascular disease

Term	Read code	Category
Pleuritic pain	1825	Non-coronary
Painful breathing -pleurodynia	1827	Non-coronary
Pleurodynia	1827-1	Non-coronary
Rib pain	182B	Non-coronary
Left subcostal pain	1973	Non-coronary
Right subcostal pain	1974	Non-coronary
Intercostal myalgia	N2410-1	Non-coronary
Costochondritis NOS	N30z8-1	Non-coronary
Tietze's disease	N336	Non-coronary
Costochondral joint syndrome	N336-1	Non-coronary
Costochondritis	N33zE	Non-coronary
Costochondritis NOS	N33zz-1	Non-coronary
[D]Painful respiration NOS	R0653	Non-coronary
[D]Pleuritic pain	R0654	Non-coronary
[D]Pleurodynia	R0655	Non-coronary
[D]Musculoskeletal chest pain	R065A	Non-coronary
[D]Non-cardiac chest pain	R065B-4	Non-coronary
[D]Non cardiac chest pain	R065B	Non-coronary
Chest pain	182	Unattributed
Central chest pain	1822	Unattributed
Precordial pain	1823	Unattributed
Anterior chest wall pain	1824	Unattributed
Parasternal pain	1826	Unattributed
Atypical chest pain	1828	Unattributed
Retrosternal pain	1829	Unattributed
Chest pain on exertion	182A	Unattributed
Costal margin chest pain	182B0	Unattributed
Chest wall pain	182C	Unattributed
Chest pain NOS	182Z	Unattributed
Seen in rapid access chest pain clinic	9N0f	Unattributed
[D]Chest pain	R065	Unattributed
[D]Precordial pain	R0651	Unattributed
[D]Anterior chest wall pain	R0652	Unattributed
[D]Chest discomfort	R0656	Unattributed
[D]Parasternal chest pain	R0659	Unattributed
[D]Chest pain NOS	R065z	Unattributed
[D]Chest pain, unspecified	R0650	Unattributed
[D]Retrosternal chest pain	R065C	Unattributed
[D]Central chest pain	R065D	Unattributed
[D] Retrosternal chest pain	R0650-1	Unattributed
[D]Chest pressure	R0657	Unattributed
[D]Chest tightness	R0658	Unattributed
[X]Other chest pain	Ryu04	Unattributed

Term	ICD 10 code	Category

Chest pain on breathing	R07.1	Non-coronary
Painful respiration	R07.1	Non-coronary
Chondrocostal junction syndrome [Tietze]	M94.0	Non-coronary
Costochondritis	M94.0	Non-coronary
Precordial pain	R07.2	Unattributed
Other chest pain	R07.3	Unattributed
Anterior chest-wall pain NOS	R07.3	Unattributed
Chest pain, unspecified	R07.4	Unattributed

**Term**	**Read code**	**Category**
H/O: angina pectoris	14A5	Angina
H/O: Angina in last year	14AJ	Angina
Frequency of angina	187	Angina
Angina self-management plan agreed	661M0	Angina
Angina self-management plan review	661N0	Angina
Angina control	662K	Angina
Angina control - good	662K0	Angina
Angina control - poor	662K1	Angina
Angina control - improving	662K2	Angina
Angina control - worsening	662K3	Angina
Angina self management plan commenced	662K4	Angina
Angina self management plan completed	662K5	Angina
Angina control NOS	662Kz	Angina
Antianginal therapy	8B27	Angina
Referral to Angina Plan self-management programme declined	8IEY	Angina
Referral to Angina Plan self-management programme	8T04	Angina
Crescendo angina	G311-1	Angina
Impending infarction	G311-2	Angina
Unstable angina	G311-3	Angina
Angina at rest	G311-4	Angina
Unstable angina	G3111	Angina
Angina at rest	G3112	Angina
Refractory angina	G3113	Angina
Worsening angina	G3114	Angina
Acute coronary insufficiency	G31y0	Angina
Angina pectoris	G33	Angina
Angina decubitus	G330	Angina
Nocturnal angina	G3300	Angina
Angina decubitus NOS	G330z	Angina
Prinzmetal's angina	G331	Angina
Variant angina pectoris	G331-1	Angina
Coronary artery spasm	G332	Angina
Angina pectoris NOS	G33z	Angina
Status anginosus	G33z0	Angina
Syncope anginosa	G33z2	Angina
Angina on effort	G33z3	Angina
Ischaemic chest pain	G33z4	Angina
New onset angina	G33z6	Angina

Stable angina	G33z7	Angina
Angina pectoris NOS	G33zz	Angina
Chronic coronary insufficiency	G34y0	Angina
Cardiac syndrome X	G37	Angina
[X]Other forms of angina pectoris	Gyu30	Angina
[RFC] Angina	HNG0012	Angina
Angina control - stable	EMISAC1	Angina
Angina control - unsatisfactory	EMISAC3	Angina
Angina grading (canadian cardiovascular society)	EMISAN1	Angina
Anginal Control: No attacks	EMISHGT65	Angina
Anginal pain	EMISCAN2	Angina
Cause of Death- Angina Pectoris	EGTON2G44	Angina
Unstable angina	EGTON458	Angina
Coronary artery bypass graft operations	792-1	CABG
Saphenous vein graft replacement of coronary artery	7920	CABG
Saphenous vein graft bypass of coronary artery	7920-1	CABG
Saphenous vein graft replacement of one coronary artery	79200	CABG
Saphenous vein graft replacement of two coronary arteries	79201	CABG
Saphenous vein graft replacement of three coronary arteries	79202	CABG
Saphenous vein graft replacement of four+ coronary arteries	79203	CABG
Saphenous vein graft replacement of coronary artery OS	7920y	CABG
Saphenous vein graft replacement coronary artery NOS	7920z	CABG
Other autograft replacement of coronary artery	7921	CABG
Other autograft bypass of coronary artery	7921-1	CABG
Autograft replacement of one coronary artery NEC	79210	CABG
Autograft replacement of two coronary arteries NEC	79211	CABG
Autograft replacement of three coronary arteries NEC	79212	CABG
Autograft replacement of four of more coronary arteries NEC	79213	CABG
Other autograft replacement of coronary artery OS	7921y	CABG
Other autograft replacement of coronary artery NOS	7921z	CABG
Allograft replacement of coronary artery	7922	CABG
Allograft bypass of coronary artery	7922-1	CABG
Allograft replacement of one coronary artery	79220	CABG
Allograft replacement of two coronary arteries	79221	CABG
Allograft replacement of three coronary arteries	79222	CABG
Allograft replacement of four or more coronary arteries	79223	CABG
Other specified allograft replacement of coronary artery	7922y	CABG
Allograft replacement of coronary artery NOS	7922z	CABG
Prosthetic replacement of coronary artery	7923	CABG
Prosthetic bypass of coronary artery	7923-1	CABG
Prosthetic replacement of one coronary artery	79230	CABG
Prosthetic replacement of two coronary arteries	79231	CABG
Prosthetic replacement of three coronary arteries	79232	CABG
Prosthetic replacement of four or more coronary arteries	79233	CABG
Prosthetic replacement of coronary artery NOS	7923z	CABG
Revision of bypass for coronary artery	7924	CABG
Revision of bypass for one coronary artery	79240	CABG
Revision of bypass for two coronary arteries	79241	CABG

Revision of bypass for three coronary arteries	79242	CABG
Revision of bypass for four or more coronary arteries	79243	CABG
Other specified revision of bypass for coronary artery	7924y	CABG
Revision of bypass for coronary artery NOS	7924z	CABG
Connection of mammary artery to coronary artery	7925	CABG
Creation of bypass from mammary artery to coronary artery	7925-1	CABG
Double anastomosis of mammary arteries to coronary arteries	79250	CABG
LIMA sequential anastomosis	79250-1	CABG
RIMA sequential anastomosis	79250-2	CABG
Double implant of mammary arteries into coronary arteries	79251	CABG
Single anast mammary art to left ant descend coronary art	79252	CABG
Single anastomosis of mammary artery to coronary artery NEC	79253	CABG
LIMA single anastomosis	79253-1	CABG
RIMA single anastomosis	79253-2	CABG
Single implantation of mammary artery into coronary artery	79254	CABG
Connection of mammary artery to coronary artery OS	7925y	CABG
Connection of mammary artery to coronary artery NOS	7925z	CABG
Connection of other thoracic artery to coronary artery	7926	CABG
Double anastom thoracic arteries to coronary arteries NEC	79260	CABG
Single anastomosis of thoracic artery to coronary artery NEC	79262	CABG
Single implantation thoracic artery into coronary artery NEC	79263	CABG
Connection of other thoracic artery to coronary artery NOS	7926z	CABG
Open angioplasty of coronary artery	79275	CABG
Other replacement of coronary artery	792C	CABG
Replacement of coronary arteries using multiple methods	792C0	CABG
Other specified replacement of coronary artery	792Cy	CABG
Replacement of coronary artery NOS	792Cz	CABG
Other bypass of coronary artery	792D	CABG
Other specified other bypass of coronary artery	792Dy	CABG
Other bypass of coronary artery NOS	792Dz	CABG
Mechanical complication of coronary bypass	SP003	CABG
Coronary artery bypass graft occlusion	SP076	CABG
[V]Presence of aortocoronary bypass graft	ZV457	CABG
[V]Presence of coronary artery bypass graft	ZV45K	CABG
[V]Presence of coronary artery bypass graft - CABG	ZV45K-1	CABG
H/O: cardiovascular disease	14A	CHD NOS
H/O: heart disease NOS	14AA	CHD NOS
H/O: Treatment for ischaemic heart disease	14AL	CHD NOS
Cardiac disease monitoring	662	CHD NOS
Heart disease monitoring	662-1	CHD NOS
CHD monitoring	662N	CHD NOS
Cardiac event recording	662Y	CHD NOS
Cardiac disease monitoring NOS	662Z	CHD NOS
Cardiovascular disease monitoring	66f	CHD NOS
Cardiovascular disease annual review	66f0	CHD NOS
Cardiovascular disease interim monitoring	66f1	CHD NOS
Coronary heart disease annual review	6A2	CHD NOS
Coronary heart disease review	6A4	CHD NOS

Repair of aneurysm of coronary artery	79271	CHD NOS
Cardiac emergency monitoring	8A51	CHD NOS
Coronary heart disease medication review	8B3k	CHD NOS
Admit ischaemic heart disease emergency	8H2V	CHD NOS
Coronary heart disease monitoring refused	8I37	CHD NOS
Exception reporting: CHD quality indicators	9h0	CHD NOS
Excepted from CHD quality indicators: Patient unsuitable	9h01	CHD NOS
Excepted from CHD quality indicators: Informed dissent	9h02	CHD NOS
Coronary heart disease monitoring administration	9Ob	CHD NOS
Attends coronary heart disease monitoring	9Ob0	CHD NOS
Refuses coronary heart disease monitoring	9Ob1	CHD NOS
Coronary heart disease monitoring default	9Ob2	CHD NOS
Coronary heart disease monitoring 1st letter	9Ob3	CHD NOS
Coronary heart disease monitoring 2nd letter	9Ob4	CHD NOS
Coronary heart disease monitoring 3rd letter	9Ob5	CHD NOS
Coronary heart disease monitoring verbal invitation	9Ob6	CHD NOS
Coronary heart disease monitoring deleted	9Ob7	CHD NOS
Coronary heart disease monitoring check done	9Ob8	CHD NOS
Coronary heart disease monitoring telephone invite	9Ob9	CHD NOS
Ischaemic heart disease	G3	CHD NOS
Arteriosclerotic heart disease	G3-1	CHD NOS
Atherosclerotic heart disease	G3-2	CHD NOS
IHD - Ischaemic heart disease	G3-3	CHD NOS
Other acute and subacute ischaemic heart disease	G31	CHD NOS
Myocardial infarction aborted	G3110	CHD NOS
MI - Myocardial infarction aborted	G3110-1	CHD NOS
Other acute and subacute ischaemic heart disease	G31y	CHD NOS
Subendocardial ischaemia	G31y2	CHD NOS
Transient myocardial ischaemia	G31y3	CHD NOS
Other acute and subacute ischaemic heart disease NOS	G31yz	CHD NOS
Other chronic ischaemic heart disease	G34	CHD NOS
Coronary atherosclerosis	G340	CHD NOS
Coronary artery disease	G340-2	CHD NOS
Ventricular cardiac aneurysm	G3410	CHD NOS
Other cardiac wall aneurysm	G3411	CHD NOS
Mural cardiac aneurysm	G3411-1	CHD NOS
Aneurysm of coronary vessels	G3412	CHD NOS
Atherosclerotic cardiovascular disease	G342	CHD NOS
Ischaemic cardiomyopathy	G343	CHD NOS
Silent myocardial ischaemia	G344	CHD NOS
Other specified chronic ischaemic heart disease	G34y	CHD NOS
Chronic myocardial ischaemia	G34y1	CHD NOS
Other specified chronic ischaemic heart disease NOS	G34yz	CHD NOS
Other chronic ischaemic heart disease NOS	G34z	CHD NOS
Asymptomatic coronary heart disease	G34z0	CHD NOS
Other specified ischaemic heart disease	G3y	CHD NOS
Ischaemic heart disease NOS	G3z	CHD NOS
Other forms of heart disease	G5	CHD NOS

Other specified heart disease	G5y	CHD NOS
Other ill-defined heart disease	G5yy	CHD NOS
Other ill-defined heart disease NOS	G5yyz	CHD NOS
Other heart disease NOS	G5yz	CHD NOS
Heart disease NOS	G5z	CHD NOS
[X]Ischaemic heart diseases	Gyu3	CHD NOS
[X]Other forms of acute ischaemic heart disease	Gyu32	CHD NOS
[X]Other forms of chronic ischaemic heart disease	Gyu33	CHD NOS
[X]Other forms of heart disease	Gyu5	CHD NOS
[RFC] Chronic heart disease (CHD)	HNG0010	CHD NOS
[RFC] Coronary heart disease	HNG0601	CHD NOS
Coronary heart disease care plan	EMISNQCO115	CHD NOS
Coronary heart disease confirmed	EMISNQCO148	CHD NOS
Coronary heart disease monitoring in primary care	EMISNQCO168	CHD NOS
Coronary heart disease monitoring in secondary care	EMISNQCO169	CHD NOS
H/O: coronary heart disease	EMISNOQFH3	CHD NOS
On coronary heart disease register	EMISNQON8	CHD NOS
Reason for influenza vaccine - chronic heart disease	EMISNQRE330	CHD NOS
CHD annual review	EMISQCH1	CHD NOS
H/O: heart failure	14A6	Heart Failure
H/O: Heart failure in last year	14AM	Heart Failure
Paroxysmal nocturnal dyspnoea	1736	Heart Failure
Suspected heart failure	1J60	Heart Failure
Heart failure confirmed	1O1	Heart Failure
O/E - pulmonary oedema	23E1	Heart Failure
New York Heart Assoc classification heart failure symptoms	388D	Heart Failure
Echocardiogram shows left ventricular systolic dysfunction	585f	Heart Failure
Echocardiogram shows left ventricular diastolic dysfunction	585g	Heart Failure
Heart failure self-management plan agreed	661M5	Heart Failure
Heart failure self-management plan review	661N5	Heart Failure
New York Heart Association Classification - Class I	662f	Heart Failure
New York Heart Association Classification - Class II	662g	Heart Failure
New York Heart Association Classification - Class III	662h	Heart Failure
New York Heart Association Classification - Class IV	662i	Heart Failure
Heart failure 6 month review	662p	Heart Failure
Congestive heart failure monitoring	662T	Heart Failure
Heart failure annual review	662W	Heart Failure
Heart failure education	679X	Heart Failure
Cardiac failure therapy	8B29	Heart Failure
Heart failure care plan discussed with patient	8CL3	Heart Failure
Admit heart failure emergency	8H2S	Heart Failure
Heart failure follow-up	8HBE	Heart Failure
Discharge from practice nurse heart failure clinic	8Hg8	Heart Failure
Referral to heart failure exercise programme	8HHz	Heart Failure
Referred to heart failure education group	8Hk0	Heart Failure
Exception reporting: LVD quality indicators	9h1	Heart Failure
Excepted from LVD quality indicators: Patient unsuitable	9h11	Heart Failure
Excepted from LVD quality indicators: Informed dissent	9h12	Heart Failure

Exception reporting: heart failure quality indicators	9hH	Heart Failure
Excepted heart failure quality indicators: Patient unsuitabl	9hH0	Heart Failure
Excepted heart failure quality indicators: Informed dissent	9hH1	Heart Failure
Seen in heart failure clinic	9N0k	Heart Failure
Seen by community heart failure nurse	9N2p	Heart Failure
Did not attend practice nurse heart failure clinic	9N4s	Heart Failure
Did not attend heart failure clinic	9N4w	Heart Failure
Referred by heart failure nurse specialist	9N6T	Heart Failure
Left ventricular dysfunction monitoring administration	9On	Heart Failure
Left ventricular dysfunction monitoring first letter	9On0	Heart Failure
Left ventricular dysfunction monitoring second letter	9On1	Heart Failure
Left ventricular dysfunction monitoring third letter	9On2	Heart Failure
Left ventricular dysfunction monitoring verbal invite	9On3	Heart Failure
Left ventricular dysfunction monitoring telephone invite	9On4	Heart Failure
Heart failure monitoring administration	9Or	Heart Failure
Heart failure review completed	9Or0	Heart Failure
Heart failure monitoring telephone invite	9Or1	Heart Failure
Heart failure monitoring verbal invite	9Or2	Heart Failure
Heart failure monitoring first letter	9Or3	Heart Failure
Heart failure monitoring second letter	9Or4	Heart Failure
Heart failure monitoring third letter	9Or5	Heart Failure
Rheumatic left ventricular failure	G1yz1	Heart Failure
Malignant hypertensive heart disease	G210	Heart Failure
Malignant hypertensive heart disease with CCF	G2101	Heart Failure
Malignant hypertensive heart disease NOS	G210z	Heart Failure
Benign hypertensive heart disease with CCF	G2111	Heart Failure
Hypertensive heart disease NOS with CCF	G21z1	Heart Failure
Hypertensive heart AND renal disease	G23	Heart Failure
Malignant hypertensive heart AND renal disease	G230	Heart Failure
Hypertensive heart&renal dis wth (congestive) heart failure	G232	Heart Failure
Hyperten heart&renal dis+both(congestv)heart and renal fail	G234	Heart Failure
Acute cor pulmonale	G400	Heart Failure
Acute pulmonary heart disease NOS	G40z	Heart Failure
Chronic cor pulmonale	G41z-1	Heart Failure
Congestive cardiomyopathy	G5540	Heart Failure
Congestive obstructive cardiomyopathy	G5540-1	Heart Failure
Heart failure	G58	Heart Failure
Cardiac failure	G58-1	Heart Failure
Congestive heart failure	G580	Heart Failure
Congestive cardiac failure	G580-1	Heart Failure
Right heart failure	G580-2	Heart Failure
Right ventricular failure	G580-3	Heart Failure
Biventricular failure	G580-4	Heart Failure
Acute congestive heart failure	G5800	Heart Failure
Chronic congestive heart failure	G5801	Heart Failure
Decompensated cardiac failure	G5802	Heart Failure
Compensated cardiac failure	G5803	Heart Failure
Congestive heart failure due to valvular disease	G5804	Heart Failure

Left ventricular failure	G581	Heart Failure
Asthma - cardiac	G581-1	Heart Failure
Pulmonary oedema - acute	G581-2	Heart Failure
Impaired left ventricular function	G581-3	Heart Failure
Acute left ventricular failure	G5810	Heart Failure
Acute heart failure	G582	Heart Failure
Heart failure with normal ejection fraction	G583	Heart Failure
HFNEF - heart failure with normal ejection fraction	G583-1	Heart Failure
Heart failure with preserved ejection fraction	G583-2	Heart Failure
Right ventricular failure	G584	Heart Failure
Heart failure NOS	G58z	Heart Failure
Cardiac failure NOS	G58z-2	Heart Failure
Left ventricular systolic dysfunction	G5yy9	Heart Failure
Left ventricular diastolic dysfunction	G5yyA	Heart Failure
Pulmonary congestion and hypostasis	H54	Heart Failure
Pulmonary congestion	H541	Heart Failure
Chronic pulmonary oedema	H5410	Heart Failure
Pulmonary oedema NOS	H541z	Heart Failure
Pulmonary congestion and hypostasis NOS	H54z	Heart Failure
Acute pulmonary oedema unspecified	H584	Heart Failure
Acute pulmonary oedema NOS	H584z	Heart Failure
Congenital cardiac failure	Q48y1	Heart Failure
[D]Cardiorespiratory failure	R2y10	Heart Failure
Heart failure as a complication of care	SP111-1	Heart Failure
AURAS-AF - consider the patient to have heart failure	EMISNQAU116	Heart Failure
Emergency heart failure admission since last appointment	EMISNQEM10	Heart Failure
Heart failure clinical pathway protocol followed	EMISNQHE59	Heart Failure
Heart failure information starter pack provided	EMISNQHE14	Heart Failure
Heart failure lifestyle plan commenced	EMISNQHE13	Heart Failure
Heart failure monitoring - co-medications	EMISNQHE20	Heart Failure
Heart failure monitoring - co-morbidities	EMISNQHE21	Heart Failure
Heart failure monitoring - multiple readmissions	EMISNQHE19	Heart Failure
Heart failure monitoring - palliative care	EMISNQHE22	Heart Failure
Heart failure monitoring - psychological issues	EMISNQHE18	Heart Failure
Heart failure monitoring - social issues	EMISNQHE17	Heart Failure
Heart failure monitoring - specialist clinical needs	EMISNQHE16	Heart Failure
Heart failure monitoring - unstable symptoms	EMISNQHE15	Heart Failure
Heart failure monitoring default	EMISNQHE72	Heart Failure
Heart failure monitoring in primary care	EMISNQHE70	Heart Failure
Heart failure monitoring in secondary care	EMISNQHE71	Heart Failure
Heart failure pathway protocol not followed	EMISNQHE58	Heart Failure
Heart failure resolved	EMISNQHE42	Heart Failure
Severe left ventricular systolic dysfunction	EMISNQSE142	Heart Failure
[RFC] Cardiac failure	HNG0013	Heart Failure
H/O: myocardial infarct <60	14A3	MI
H/O: myocardial infarct >60	14A4	MI
H/O: Myocardial infarction in last year	14AH	MI
History of myocardial infarction	14AT	MI

ECG: myocardial infarction	323	MI
ECG: old myocardial infarction	3232	MI
ECG: antero-septal infarct.	3233	MI
ECG:posterior/inferior infarct	3234	MI
ECG: subendocardial infarct	3235	MI
ECG: lateral infarction	3236	MI
ECG: myocardial infarct NOS	323Z	MI
Diab mellit insulin-glucose infus acute myocardial infarct	889A	MI
Acute myocardial infarction	G30	MI
Attack - heart	G30-1	MI
Coronary thrombosis	G30-2	MI
Cardiac rupture following myocardial infarction (MI)	G30-3	MI
Heart attack	G30-4	MI
MI - acute myocardial infarction	G30-5	MI
Thrombosis - coronary	G30-6	MI
Silent myocardial infarction	G30-7	MI
Acute anterolateral infarction	G300	MI
Other specified anterior myocardial infarction	G301	MI
Acute anteroapical infarction	G3010	MI
Acute anteroseptal infarction	G3011	MI
Anterior myocardial infarction NOS	G301z	MI
Acute inferolateral infarction	G302	MI
Acute inferoposterior infarction	G303	MI
Posterior myocardial infarction NOS	G304	MI
Lateral myocardial infarction NOS	G305	MI
True posterior myocardial infarction	G306	MI
Acute subendocardial infarction	G307	MI
Acute non-Q wave infarction	G3070	MI
Acute non-ST segment elevation myocardial infarction	G3071	MI
Inferior myocardial infarction NOS	G308	MI
Acute Q-wave infarct	G309	MI
Acute posterolateral myocardial infarction	G30B	MI
Acute transmural myocardial infarction of unspecif site	G30X	MI
Acute ST segment elevation myocardial infarction	G30X0	MI
Other acute myocardial infarction	G30y	MI
Acute atrial infarction	G30y0	MI
Acute papillary muscle infarction	G30y1	MI
Acute septal infarction	G30y2	MI
Other acute myocardial infarction NOS	G30yz	MI
Acute myocardial infarction NOS	G30z	MI
Postmyocardial infarction syndrome	G310	MI
Dressler's syndrome	G310-1	MI
Acute coronary syndrome	G3115	MI
Microinfarction of heart	G31y1	MI
Old myocardial infarction	G32	MI
Healed myocardial infarction	G32-1	MI
Personal history of myocardial infarction	G32-2	MI
Post infarct angina	G33z5	MI

Subsequent myocardial infarction	G35	MI
Subsequent myocardial infarction of anterior wall	G350	MI
Subsequent myocardial infarction of inferior wall	G351	MI
Subsequent myocardial infarction of other sites	G353	MI
Subsequent myocardial infarction of unspecified site	G35X	MI
Certain current complication follow acute myocardial infarct	G36	MI
Haemopericardium/current comp folow acut myocard infarct	G360	MI
Atrial septal defect/curr comp folow acut myocardal infarct	G361	MI
Ventric septal defect/curr comp fol acut myocardal infarctn	G362	MI
Ruptur cardiac wall w'out haemopericard/cur comp fol ac MI	G363	MI
Ruptur chordae tendinae/curr comp fol acute myocard infarct	G364	MI
Rupture papillary muscle/curr comp fol acute myocard infarct	G365	MI
Thrombosis atrium,auric append&vent/curr comp foll acute MI	G366	MI
Postoperative myocardial infarction	G38	MI
Postoperative transmural myocardial infarction anterior wall	G380	MI
Postoperative transmural myocardial infarction inferior wall	G381	MI
Postoperative transmural myocardial infarction unspec site	G383	MI
Postoperative subendocardial myocardial infarction	G384	MI
Postoperative myocardial infarction, unspecified	G38z	MI
Post infarction pericarditis	G501	MI
[X]Acute transmural myocardial infarction of unspecif site	Gyu34	MI
[X]Subsequent myocardial infarction of other sites	Gyu35	MI
[X]Subsequent myocardial infarction of unspecified site	Gyu36	MI
[RFC] Myocardial infarction (MI)	HNG0009	MI
Cause of Death- Acute Myocardial Infarction	EGTON2G41	MI
Cause of Death- Myocardial Infarction		MI
First myocardial infarction	EMISR4QFI1	MI
H/O: aortic aneurysm	14AE	PAD AAA
H/O: Peripheral vascular disease procedure	14NB	PAD AAA
Ischaemic toe	2G63	PAD AAA
Femoral arteriogram abnormal	5593	PAD AAA
Lower limb arteriogram abnorm.	55A2	PAD AAA
Aortic aneurysm monitoring	66f3	PAD AAA
Emerg aortic bypass by anastomosis axillary to femoral art	7A100	PAD AAA
Bypass aorta by anastomosis axillary to femoral artery NEC	7A101	PAD AAA
Axillo-bifemoral bypass graft	7A102	PAD AAA
Axillo-unifemoral PTFE bypass graft	7A103	PAD AAA
Emerg repl aneurysm bifurc aorta by anast aorta to fem art	7A110	PAD AAA
Emerg repl aneurysm bifurc aorta by anast aorta to iliac a	7A112	PAD AAA
Y graft of abdominal Aortic aneurysm (emergency)	7A112-1	PAD AAA
Other bypass of bifurcation of aorta	7A12	PAD AAA
Emerg bypass bifurc aorta by anast aorta to femoral artery	7A120	PAD AAA
Bypass bifurc aorta by anastom aorta to femoral artery NEC	7A121	PAD AAA
Aorto bifemoral graft	7A121-1	PAD AAA
Dacron aortofemoral Y graft	7A121-2	PAD AAA
Bypass bifurcation aorta by anastom aorta to iliac artery	7A123	PAD AAA
Aorto biiliac graft	7A123-1	PAD AAA
Dacron aortoiliac Y graft	7A123-2	PAD AAA

Other specified other bypass of bifurcation of aorta	7A12y	PAD AAA
Other bypass of bifurcation of aorta NOS	7A12z	PAD AAA
Emergency replacement of aneurysmal segment of aorta	7A13	PAD AAA
Emergency repair of aortic aneurysm	7A13-1	PAD AAA
Emerg replace aneurysm asc aorta by anastom aorta to aorta	7A130	PAD AAA
Emerg replace aneurysm thor aorta by anastom aorta to aorta	7A131	PAD AAA
Emerg replace aneurysm infrarenal aorta by anast aorta/aorta	7A133	PAD AAA
Emerg replace aneurysm abdom aorta by anast aorta/aorta NEC	7A134	PAD AAA
Tube graft abdominal Aortic aneurysm (emergency)	7A134-1	PAD AAA
Emergency replacement of aneurysmal segment of aorta OS	7A13y	PAD AAA
Emergency replacement of aneurysmal segment of aorta NOS	7A13z	PAD AAA
Open embolectomy of bifurcation of aorta	7A192	PAD AAA
Other bypass of iliac artery	7A41	PAD AAA
Other bypass of iliac artery by anastomosis	7A41-1	PAD AAA
Emerg bypass iliac art by iliac/femoral art anastomosis NEC	7A410	PAD AAA
Bypass iliac artery by iliac/femoral artery anastomosis NEC	7A411	PAD AAA
Emerg bypass iliac artery by femoral/femoral art anast NEC	7A412	PAD AAA
Emergency femoro-femoral prosthetic cross over graft	7A412-1	PAD AAA
Bypass iliac artery by femoral/femoral art anastomosis NEC	7A413	PAD AAA
Femoro-femoral prosthetic cross over graft	7A413-1	PAD AAA
Emerg bypass comm iliac art by aorta/com iliac art anast NEC	7A414	PAD AAA
Emerg bypass leg artery by aorta/com fem art anastomosis NEC	7A416	PAD AAA
Bypass common iliac artery by aorta/com iliac art anast NEC	7A419	PAD AAA
Bypass leg artery by aorta/com femoral art anastomosis NEC	7A41B	PAD AAA
Bypass leg artery by aorta/deep femoral art anastomosis NEC	7A41C	PAD AAA
Bypass iliac artery by iliac/iliac artery anastomosis NEC	7A41D	PAD AAA
Emergency bypass of iliac artery by unspecified anastomosis	7A41E	PAD AAA
Ilio-femoral prosthetic cross over graft	7A41F	PAD AAA
Other specified other bypass of iliac artery	7A41y	PAD AAA
Other bypass of iliac artery NOS	7A41z	PAD AAA
Reconstruction of iliac artery	7A42	PAD AAA
Reconstruction of common iliac artery	7A42-1	PAD AAA
Endarterectomy and patch repair of iliac artery	7A420	PAD AAA
Endarterectomy and patch repair of common iliac artery	7A420-1	PAD AAA
Iliac endarterectomy and patch	7A420-2	PAD AAA
Endarterectomy of iliac artery NEC	7A421	PAD AAA
Endarterectomy of common iliac artery NEC	7A421-1	PAD AAA
Other specified reconstruction of iliac artery	7A42y	PAD AAA
Reconstruction of iliac artery NOS	7A42z	PAD AAA
Other open operations on iliac artery	7A43	PAD AAA
Other open operations on common iliac artery	7A43-1	PAD AAA
Repair of iliac artery NEC	7A430	PAD AAA
Repair of common iliac artery NEC	7A430-1	PAD AAA
Open embolectomy of iliac artery	7A431	PAD AAA
Open embolectomy of common iliac artery	7A431-1	PAD AAA
Open insertion of iliac artery stent	7A433	PAD AAA
Percutaneous transluminal angioplasty of iliac artery	7A440	PAD AAA
Percutaneous transluminal embolectomy of iliac artery	7A441	PAD AAA

Insertion of iliac artery stent	7A443	PAD AAA
Percutaneous transluminal insertion of iliac artery stent	7A444	PAD AAA
Other specified transluminal operation on iliac artery	7A44y	PAD AAA
Transluminal operation on iliac artery NOS	7A44z	PAD AAA
Other emergency bypass of femoral artery or popliteal artery	7A47	PAD AAA
Other emerg bypass femoral or popliteal art by anastomosis	7A47-1	PAD AAA
Other emergency bypass of common femoral artery	7A47-2	PAD AAA
Other emergency bypass of deep femoral artery	7A47-3	PAD AAA
Other emergency bypass of popliteal artery	7A47-4	PAD AAA
Other emergency bypass of superficial femoral artery	7A47-5	PAD AAA
Other emergency bypass of femoral artery	7A47-6	PAD AAA
Emerg bypass femoral art by fem/pop art anast c prosth NEC	7A470	PAD AAA
Emerg bypass popliteal art by pop/pop art anast c prosth NEC	7A471	PAD AAA
Emerg bypass femoral art by fem/pop a anast c vein graft NEC	7A472	PAD AAA
Emerg bypass pop art by pop/pop art anast c vein graft NEC	7A473	PAD AAA
Emerg bypass femoral art by fem/tib art anast c prosth NEC	7A474	PAD AAA
Emerg bypass femoral art by fem/tib a anast c vein graft NEC	7A476	PAD AAA
Emerg bypass pop art by pop/tib art anast c vein graft NEC	7A477	PAD AAA
Emerg bypass popliteal art by pop/peron a anast c prosth NEC	7A479	PAD AAA
Emerg bypass fem art by fem/peron a anast c vein graft NEC	7A47A	PAD AAA
Emerg bypass pop art by pop/peron art anast c vein graft NEC	7A47B	PAD AAA
Emerg bypass femoral artery by fem/fem art anastomosis NEC	7A47C	PAD AAA
Emerg bypass popliteal artery by pop/fem art anastomosis NEC	7A47D	PAD AAA
Other emergency bypass of femoral or popliteal artery OS	7A47y	PAD AAA
Other emergency bypass of femoral or popliteal artery NOS	7A47z	PAD AAA
Other bypass of femoral artery or popliteal artery	7A48	PAD AAA
Other bypass of femoral or popliteal artery by anastomosis	7A48-1	PAD AAA
Other bypass of common femoral artery	7A48-2	PAD AAA
Other bypass of femoral artery	7A48-4	PAD AAA
Other bypass of popliteal artery	7A48-5	PAD AAA
Other bypass of superficial femoral artery	7A48-6	PAD AAA
Bypass femoral artery by fem/pop art anast c prosthesis NEC	7A480	PAD AAA
Bypass popliteal artery by pop/pop a anast c prosthesis NEC	7A481	PAD AAA
Bypass femoral artery by fem/pop art anast c vein graft NEC	7A482	PAD AAA
Bypass popliteal artery by pop/pop a anast c vein graft NEC	7A483	PAD AAA
Bypass femoral artery by fem/tib art anast c prosthesis NEC	7A484	PAD AAA
Bypass popliteal artery by pop/tib a anast c prosthesis NEC	7A485	PAD AAA
Bypass femoral artery by fem/tib art anast c vein graft NEC	7A486	PAD AAA
Bypass popliteal artery by pop/tib a anast c vein graft NEC	7A487	PAD AAA
Bypass femoral artery by fem/peron a anast c prosthesis NEC	7A488	PAD AAA
Bypass popliteal artery by pop/peron art anast c prosth NEC	7A489	PAD AAA
Bypass femoral artery by fem/peron a anast c vein graft NEC	7A48A	PAD AAA
Bypass popliteal art by pop/peron art anast c vein graft NEC	7A48B	PAD AAA
Bypass femoral artery by femoral/femoral art anastomosis NEC	7A48C	PAD AAA
Bypass popliteal artery by pop/fem artery anastomosis NEC	7A48D	PAD AAA
Femoro-femoral prosthetic cross over graft	7A48E	PAD AAA
Other bypass of femoral artery or popliteal artery OS	7A48y	PAD AAA
Other bypass of femoral artery or popliteal artery NOS	7A48z	PAD AAA

Reconstruction of femoral artery or popliteal artery	7A49	PAD AAA
Reconstruction of common femoral artery	7A49-1	PAD AAA
Reconstruction of deep femoral artery	7A49-2	PAD AAA
Reconstruction of femoral artery	7A49-3	PAD AAA
Reconstruction of popliteal artery	7A49-4	PAD AAA
Reconstruction of superficial femoral artery	7A49-5	PAD AAA
Endarterectomy and patch repair of femoral artery	7A490	PAD AAA
Endarterectomy and patch repair of popliteal artery	7A491	PAD AAA
Endarterectomy of femoral artery NEC	7A492	PAD AAA
Endarterectomy of popliteal artery NEC	7A493	PAD AAA
Profundoplasty femoral artery & patch repair deep fem artery	7A494	PAD AAA
Profundoplasty and patch repair of popliteal artery	7A495	PAD AAA
Profundoplasty of femoral artery NEC	7A496	PAD AAA
Profundoplasty of popliteal artery NEC	7A497	PAD AAA
Reconstruction of femoral artery with vein graft	7A498	PAD AAA
Reconstruction of popliteal artery with vein graft	7A499	PAD AAA
Reconstruction of femoral or popliteal artery OS	7A49y	PAD AAA
Reconstruction of femoral or popliteal artery NOS	7A49z	PAD AAA
Other open operations on femoral artery or popliteal artery	7A4A	PAD AAA
Other open operations on common femoral artery	7A4A-1	PAD AAA
Other open operations on deep femoral artery	7A4A-2	PAD AAA
Other open operations on popliteal artery	7A4A-3	PAD AAA
Other open operations on superficial femoral artery	7A4A-4	PAD AAA
Repair of femoral artery NEC	7A4A0	PAD AAA
Repair of popliteal artery NEC	7A4A1	PAD AAA
Open embolectomy of femoral artery	7A4A2	PAD AAA
Open thrombectomy of femoral artery	7A4A2-1	PAD AAA
Open femoral embolectomy	7A4A2-2	PAD AAA
Open embolectomy popliteal artery	7A4A3	PAD AAA
Open thrombectomy of popliteal artery	7A4A3-1	PAD AAA
Ligation of aneurysm of popliteal artery	7A4A4	PAD AAA
Operation on aneurysm of femoral artery NEC	7A4A5	PAD AAA
Operation on popliteal artery NEC	7A4A6	PAD AAA
Repair of femoral artery with temporary silastic shunt	7A4A7	PAD AAA
Repair of popliteal artery with temporary silastic shunt	7A4A8	PAD AAA
Other open operation on femoral or popliteal artery OS	7A4Ay	PAD AAA
Other open operation on femoral or popliteal artery NOS	7A4Az	PAD AAA
Percutaneous transluminal angioplasty of femoral artery	7A4B0	PAD AAA
Percutaneous transluminal angioplasty of popliteal artery	7A4B1	PAD AAA
Percutaneous transluminal embolectomy of femoral artery	7A4B2	PAD AAA
Percutaneous transluminal embolectomy of popliteal artery	7A4B3	PAD AAA
Percutaneous transluminal embolisation of femoral artery	7A4B4	PAD AAA
Percutaneous transluminal embolisation of popliteal artery	7A4B5	PAD AAA
Percut translum thrombolysis femoral graft streptokinase	7A4B8	PAD AAA
Percutaneous transluminal insertion of stent femoral artery	7A4B9	PAD AAA
Revision of reconstruction of artery	7A50	PAD AAA
Revision of reconstruction involving aorta	7A500	PAD AAA
Revision of reconstruction involving iliac artery	7A501	PAD AAA

Revision of reconstruction involving femoral artery	7A502	PAD AAA
Revision of reconstruction of popliteal artery	7A503	PAD AAA
Other specified revision of reconstruction of artery	7A50y	PAD AAA
Revision of reconstruction of artery NOS	7A50z	PAD AAA
Gas gangrene-foot	A3A0F	PAD AAA
Diabetes mellitus with peripheral circulatory disorder	C107	PAD AAA
Diabetes mellitus, juvenile +peripheral circulatory disorder	C1070	PAD AAA
Diabetes mellitus, adult, + peripheral circulatory disorder	C1071	PAD AAA
IDDM with peripheral circulatory disorder	C1073	PAD AAA
NIDDM with peripheral circulatory disorder	C1074	PAD AAA
Other specified diabetes mellitus with periph circ comps	C107y	PAD AAA
Diabetes mellitus NOS with peripheral circulatory disorder	C107z	PAD AAA
Insulin dependent diab mell with peripheral angiopathy	C108G	PAD AAA
Non-insulin-dependent d m with peripheral angiopath	C109F	PAD AAA
Type II diabetes mellitus with peripheral angiopathy	C109F-1	PAD AAA
Type 2 diabetes mellitus with peripheral angiopathy	C109F-2	PAD AAA
Type 1 diabetes mellitus with peripheral angiopathy	C10EG	PAD AAA
Type 2 diabetes mellitus with peripheral angiopathy	C10FF	PAD AAA
Aorto-iliac disease	G700-1	PAD AAA
Extremity artery atheroma	G702	PAD AAA
Extremity artery atheroma NOS	G702z	PAD AAA
Aortic aneurysm	G71	PAD AAA
Abdominal aortic aneurysm which has ruptured	G713	PAD AAA
Ruptured abdominal aortic aneurysm	G713-1	PAD AAA
Ruptured suprarenal aortic aneurysm	G7130	PAD AAA
Abdominal aortic aneurysm without mention of rupture	G714	PAD AAA
AAA - Abdominal aortic aneurysm without mention of rupture	G714-1	PAD AAA
Juxtarenal aortic aneurysm	G7140	PAD AAA
Ruptured aortic aneurysm NOS	G715	PAD AAA
Thoracoabdominal aortic aneurysm, ruptured	G7150	PAD AAA
Aortic aneurysm without mention of rupture NOS	G716	PAD AAA
Thoracoabdominal aortic aneurysm, without mention of rupture	G7160	PAD AAA
Leaking abdominal aortic aneurysm	G718	PAD AAA
Aortic aneurysm NOS	G71z	PAD AAA
Other peripheral vascular disease	G73	PAD AAA
Peripheral ischaemic vascular disease	G73-1	PAD AAA
Ischaemia of legs	G73-2	PAD AAA
Peripheral ischaemia	G73-3	PAD AAA
Thromboangiitis obliterans	G731	PAD AAA
Buerger's disease	G7310	PAD AAA
Thromboangiitis obliterans NOS	G731z	PAD AAA
Peripheral gangrene	G732	PAD AAA
Gangrene of toe	G7320	PAD AAA
Gangrene of foot	G7321	PAD AAA
Ischaemic foot	G733	PAD AAA
Other specified peripheral vascular disease	G73y	PAD AAA
Diabetic peripheral angiopathy	G73y0	PAD AAA
Peripheral angiopathic disease EC NOS	G73y1	PAD AAA

Other specified peripheral vascular disease NOS	G73yz	PAD AAA
Peripheral vascular disease NOS	G73z	PAD AAA
Intermittent claudication	G73z0	PAD AAA
Claudication	G73z0-1	PAD AAA
Peripheral vascular disease NOS	G73zz	PAD AAA
Aortoiliac obstruction	G740-2	PAD AAA
Embolism and thrombosis of the femoral artery	G7424	PAD AAA
Embolism and thrombosis of the popliteal artery	G7425	PAD AAA
Embolism and thrombosis of the anterior tibial artery	G7426	PAD AAA
Embolism and thrombosis of the dorsalis pedis artery	G7427	PAD AAA
Embolism and thrombosis of a leg artery NOS	G7429	PAD AAA
Peripheral arterial embolism and thrombosis NOS	G742z	PAD AAA
Embolism and/or thrombosis of the common iliac artery	G74y0	PAD AAA
Embolism and/or thrombosis of the internal iliac artery	G74y1	PAD AAA
Embolism and/or thrombosis of the external iliac artery	G74y2	PAD AAA
Embolism and thrombosis of the iliac artery unspecified	G74y3	PAD AAA
[X]Other specified peripheral vascular diseases	Gyu74	PAD AAA
Ischaemic leg ulcer	M271-2	PAD AAA
Ischaemic ulcer diabetic foot	M2710	PAD AAA
Arterial leg ulcer	M2713	PAD AAA
Mixed venous and arterial leg ulcer	M2714	PAD AAA
[D]Gangrene of toe in diabetic	R0542	PAD AAA
[D]Widespread diabetic foot gangrene	R0543	PAD AAA
[D]Failure of peripheral circulation	R0550	PAD AAA
[D]Peripheral circulatory failure	R0550-1	PAD AAA
[RFC] Peripheral vascular disease	HNG0172	PAD AAA
Ischaemic foot	EMISNQIS4	PAD AAA
Peripheral vascular disease annual review	EMISNQPE9	PAD AAA
Peripheral vascular disease monitoring administration	EMISNQPE10	PAD AAA
Peripheral vascular disease monitoring first letter	EMISNQPE11	PAD AAA
Peripheral vascular disease monitoring second letter	EMISNQPE12	PAD AAA
Peripheral vascular disease monitoring third letter	EMISNQPE13	PAD AAA
Transluminal balloon angioplasty of coronary artery	7928	PCI
Percutaneous balloon coronary angioplasty	7928-1	PCI
Percut transluminal balloon angioplasty one coronary artery	79280	PCI
Percut translum balloon angioplasty mult coronary arteries	79281	PCI
Percut translum balloon angioplasty bypass graft coronary a	79282	PCI
Percut translum cutting balloon angioplasty coronary artery	79283	PCI
Transluminal balloon angioplasty of coronary artery OS	7928y	PCI
Transluminal balloon angioplasty of coronary artery NOS	7928z	PCI
Percutaneous transluminal laser coronary angioplasty	79290	PCI
Rotary blade coronary angioplasty	79293	PCI
Insertion of coronary artery stent	79294	PCI
Insertion of drug-eluting coronary artery stent	79295	PCI
Percutaneous transluminal atherectomy of coronary artery	79296	PCI
Endarterectomy of coronary artery NEC	792B0	PCI
Perc translumin balloon angioplasty stenting coronary artery	793G	PCI
Perc translum ball angio insert 1-2 drug elut stents cor art	793G0	PCI

Perc tran ball angio ins 3 or more drug elut stents cor art	793G1	PCI
Perc translum balloon angioplasty insert 1-2 stents cor art	793G2	PCI
Percutaneous cor balloon angiop 3 more stents cor art NEC	793G3	PCI
OS perc translumina balloon angioplast stenting coronary art	793Gy	PCI
Perc translum balloon angioplasty stenting coronary art NOS	793Gz	PCI
Percutaneous transluminal angioplasty of artery NEC	7A540	PCI
Rotary blade angioplasty	7A545	PCI
Percutaneous transluminal atherectomy	7A548	PCI
Percutaneous transluminal balloon angioplasty of artery	7A564	PCI
Peroperative angioplasty	7A6G1	PCI
Prosthetic graft patch angioplasty	7A6H3	PCI
Percutaneous transluminal angioplasty of vascular graft	7A6H4	PCI
[V]Presence of coronary angioplasty implant and graft	ZV458	PCI
[V]Status following coronary angioplasty NOS	ZV45L	PCI
Stroke group member	13YA	Stroke
H/O: CVA/stroke	14A7	Stroke
H/O: CVA	14A7-1	Stroke
H/O: stroke	14A7-2	Stroke
H/O: TIA	14AB	Stroke
H/O: Stroke in last year	14AK	Stroke
Stroke self-management plan agreed	661M7	Stroke
Stroke self-management plan review	661N7	Stroke
Stroke/CVA annual review	662e	Stroke
Stroke annual review	6.62E+01	Stroke
Stroke 6 month review	662M1	Stroke
Stroke initial post discharge review	662M2	Stroke
Haemorrhagic stroke monitoring	662o	Stroke
Evacuation of subdural haematoma	70170	Stroke
Evacuation of extradural haematoma	70320	Stroke
Delivery of rehabilitation for stroke	7P242	Stroke
Stroke / transient ischaemic attack referral	8HBJ	Stroke
Ref to multidisciplinary stroke function improvement service	8HHM	Stroke
Exception reporting: stroke quality indicators	9h2	Stroke
Excepted from stroke quality indicators: Patient unsuitable	9h21	Stroke
Excepted from stroke quality indicators: Informed dissent	9h22	Stroke
Rupture of syphilitic cerebral aneurysm	A94y6	Stroke
Mitochond encephalopathy, lact acidosis & strokelike episode	C3151	Stroke
[X]Other transient cerebral ischaemic attacks and related syndromes	Fyu55	Stroke
[X]Other lacunar syndromes	Fyu56	Stroke
[X]Other vascular syndroms/brain in cerebrovasculr diseases	Fyu57	Stroke
Subarachnoid haemorrhage	G60	Stroke
Ruptured berry aneurysm	G600	Stroke
Subarachnoid haemorrhage from carotid siphon and bifurcation	G601	Stroke
Subarachnoid haemorrhage from middle cerebral artery	G602	Stroke
Subarachnoid haemorrhage from anterior communicating artery	G603	Stroke
Subarachnoid haemorrhage from posterior communicating artery	G604	Stroke
Subarachnoid haemorrhage from basilar artery	G605	Stroke

Subarachnoid haemorrhage from vertebral artery	G606	Stroke
Subarachnoid haemorrh from intracranial artery, unspecif	G60X	Stroke
Subarachnoid haemorrhage NOS	G60z	Stroke
Intracerebral haemorrhage	G61	Stroke
CVA - cerebrovascular accid due to intracerebral haemorrhage	G61-1	Stroke
Stroke due to intracerebral haemorrhage	G61-2	Stroke
Cortical haemorrhage	G610	Stroke
Internal capsule haemorrhage	G611	Stroke
Basal nucleus haemorrhage	G612	Stroke
Cerebellar haemorrhage	G613	Stroke
Pontine haemorrhage	G614	Stroke
Bulbar haemorrhage	G615	Stroke
External capsule haemorrhage	G616	Stroke
Intracerebral haemorrhage, intraventricular	G617	Stroke
Intracerebral haemorrhage, multiple localized	G618	Stroke
Lobar cerebral haemorrhage	G619	Stroke
Intracerebral haemorrhage in hemisphere, unspecified	G61X	Stroke
Left sided intracerebral haemorrhage, unspecified	G61X0	Stroke
Right sided intracerebral haemorrhage, unspecified	G61X1	Stroke
Intracerebral haemorrhage NOS	G61z	Stroke
Other and unspecified intracranial haemorrhage	G62	Stroke
Extradural haemorrhage - nontraumatic	G620	Stroke
Subdural haemorrhage - nontraumatic	G621	Stroke
Subdural haematoma - nontraumatic	G622	Stroke
Subdural haemorrhage NOS	G623	Stroke
Intracranial haemorrhage NOS	G62z	Stroke
Cerebral infarct due to thrombosis of precerebral arteries	G63y0	Stroke
Cerebral infarction due to embolism of precerebral arteries	G63y1	Stroke
Cerebral arterial occlusion	G64	Stroke
CVA - cerebral artery occlusion	G64-1	Stroke
Infarction - cerebral	G64-2	Stroke
Stroke due to cerebral arterial occlusion	G64-3	Stroke
Cerebral thrombosis	G640	Stroke
Cerebral infarction due to thrombosis of cerebral arteries	G6400	Stroke
Cerebral embolism	G641	Stroke
Cerebral embolus	G641-1	Stroke
Cerebral infarction due to embolism of cerebral arteries	G6410	Stroke
Cerebral infarction NOS	G64z	Stroke
Brainstem infarction NOS	G64z-1	Stroke
Cerebellar infarction	G64z-2	Stroke
Brainstem infarction	G64z0	Stroke
Wallenberg syndrome	G64z1	Stroke
Lateral medullary syndrome	G64z1-1	Stroke
Left sided cerebral infarction	G64z2	Stroke
Right sided cerebral infarction	G64z3	Stroke
Infarction of basal ganglia	G64z4	Stroke
Transient cerebral ischaemia	G65	Stroke
Transient ischaemic attack	G65-2	Stroke

Carotid artery syndrome hemispheric	G653	Stroke
Multiple and bilateral precerebral artery syndromes	G654	Stroke
Carotid territory transient ischaemic attack	G657	Stroke
Other transient cerebral ischaemia	G65y	Stroke
Transient cerebral ischaemia NOS	G65z	Stroke
Impending cerebral ischaemia	G65z0	Stroke
Intermittent cerebral ischaemia	G65z1	Stroke
Transient cerebral ischaemia NOS	G65zz	Stroke
Stroke and cerebrovascular accident unspecified	G66	Stroke
CVA unspecified	G66-1	Stroke
Stroke unspecified	G66-2	Stroke
CVA - Cerebrovascular accident unspecified	G66-3	Stroke
Middle cerebral artery syndrome	G660	Stroke
Anterior cerebral artery syndrome	G661	Stroke
Posterior cerebral artery syndrome	G662	Stroke
Brain stem stroke syndrome	G663	Stroke
Cerebellar stroke syndrome	G664	Stroke
Pure motor lacunar syndrome	G665	Stroke
Pure sensory lacunar syndrome	G666	Stroke
Left sided CVA	G667	Stroke
Right sided CVA	G668	Stroke
Generalised ischaemic cerebrovascular disease NOS	G671	Stroke
Acute cerebrovascular insufficiency NOS	G6710	Stroke
Chronic cerebral ischaemia	G6711	Stroke
Generalised ischaemic cerebrovascular disease NOS	G671z	Stroke
Cereb infarct due cerebral venous thrombosis, nonpyogenic	G6760	Stroke
Late effects of cerebrovascular disease	G68	Stroke
Sequelae of subarachnoid haemorrhage	G680	Stroke
Sequelae of intracerebral haemorrhage	G681	Stroke
Sequelae of other nontraumatic intracranial haemorrhage	G682	Stroke
Sequelae of cerebral infarction	G683	Stroke
Sequelae/other + unspecified cerebrovascular diseases	G68W	Stroke
Sequelae of stroke,not specfd as h'morrhage or infarction	G68X	Stroke
Cereb infarct due unsp occlus/stenos precerebr arteries	G6W	Stroke
Cerebrl infarctn due/unspcf occlusn or sten/cerebrl artrs	G6X	Stroke
[X]Subarachnoid haemorrhage from other intracranial arteries	Gyu60	Stroke
[X]Other subarachnoid haemorrhage	Gyu61	Stroke
[X]Other intracerebral haemorrhage	Gyu62	Stroke
[X]Cerebrl infarctn due/unspcf occlusn or sten/cerebrl artrs	Gyu63	Stroke
[X]Other cerebral infarction	Gyu64	Stroke
[X]Occlusion and stenosis of other precerebral arteries	Gyu65	Stroke
[X]Occlusion and stenosis of other cerebral arteries	Gyu66	Stroke
[X]Sequelae of stroke,not specfd as h'morrhage or infarction	Gyu6C	Stroke
[X]Subarachnoid haemorrh from intracranial artery, unspecif	Gyu6E	Stroke
[X]Intracerebral haemorrhage in hemisphere, unspecified	Gyu6F	Stroke
[X]Cereb infarct due unsp occlus/stenos precerebr arteries	Gyu6G	Stroke
CVA - cerebrovascular accident in the puerperium	L440-1	Stroke
Stroke in the puerperium	L440-2	Stroke

[V]Personal history of stroke	ZV125-1	Stroke
[V]Personal history of cerebrovascular accident (CVA)	ZV125-2	Stroke
[RFC] Ischaemic attack	HNG0237	Stroke
[RFC] Stroke	HNG0235	Stroke
[RFC] Stroke	HNG0602	Stroke
[RFC] Stroke/CVA	HNG0234	Stroke
Central post-stroke pain	EMISNQCE10	Stroke
Discharge from community stroke service	EMISNQDI251	Stroke
Referral to community stroke service	EMISNQRE623	Stroke
Referral to stroke rehabilitation service	EMISNQRE602	Stroke
Suspected transient ischaemic attack	EMISNQSU26	Stroke
Cerebral infarction with haemorrhagic transformation	EMISNQCE4	Stroke
Cause of Death- Cerebral Infarct		Stroke
[RFC] CVA	HNGP003	Stroke
H/O ventricular fibrillation	14AD	VA Cardiac arrest/death
O/E - collapse -cardiac arrest	2241	VA Cardiac arrest/death
ECG: ventricular fibrillation	3283	VA Cardiac arrest/death
Cardiac massage - open	79321-1	VA Cardiac arrest/death
Advanced cardiopulmonary resuscitation	7L1H6	VA Cardiac arrest/death
Cardiac massage - external	853	VA Cardiac arrest/death
Closed cardiac massage alone	8531	VA Cardiac arrest/death
Closed cardiac massage+ventil.	8532	VA Cardiac arrest/death
Cardiopulmonary resuscitation	8532-1	VA Cardiac arrest/death
External cardiac massage NOS	853Z	VA Cardiac arrest/death
Ventricular fibrillation and flutter	G574	VA Cardiac arrest/death
Ventricular fibrillation	G5740	VA Cardiac arrest/death
Cardiac arrest-ventricular fibrillation	G5740-1	VA Cardiac arrest/death
Ventricular fibrillation and flutter NOS	G574z	VA Cardiac arrest/death
Cardiac arrest	G575	VA Cardiac arrest/death
Cardio-respiratory arrest	G575-1	VA Cardiac arrest/death
Asystole	G575-2	VA Cardiac arrest/death
Cardiac arrest with successful resuscitation	G5750	VA Cardiac arrest/death
Sudden cardiac death, so described	G5751	VA Cardiac arrest/death
Electromechanical dissociation with successful resuscitation	G5752	VA Cardiac arrest/death
Electromechanical dissociation	G5753	VA Cardiac arrest/death
Cardiac arrest, unspecified	G575z	VA Cardiac arrest/death
Cardiac arrest as a complication of care	SP110	VA Cardiac arrest/death

**Term**	**ICD 10 code**	**Category**
Angina pectoris	I20	Angina
Unstable angina	I200	Angina
Angina pectoris with documented spasm	I201	Angina
Other forms of angina pectoris	I208	Angina
Angina pectoris unspecified	I209	Angina
Presence of coronary angioplasty implant and graft	Z955	CABG
Other acute ischaemic heart diseases	I24	CHD NOS
Coronary thrombosis not resulting in myocardial infarction	I240	CHD NOS
Other forms of acute ischaemic heart disease	I248	CHD NOS

Acute ischaemic heart disease, unspecified	I249	CHD NOS
Chronic ischaemic heart disease	I25	CHD NOS
Atherosclerotic cardiovascular disease, so described	I250	CHD NOS
Atherosclerotic heart disease	I251	CHD NOS
Coronary artery aneurysm	I254	CHD NOS
Ischaemic cardiomyopathy	I255	CHD NOS
Silent myocardial ischaemia	I256	CHD NOS
Other forms of chronic ischaemic heart disease	I258	CHD NOS
Chronic ischaemic heart disease, unspecified	I259	CHD NOS
Hypertensive heart disease with (congestive) heart failure	I110	Heart Failure
Hypertensive heart and renal disease with (congestive) heart failure	I130	Heart Failure
Hypertensive heart and renal disease with both (congestive) heart failure and renal failure	I132	Heart Failure
Heart failure	I50	Heart Failure
Congestive heart failure	I500	Heart Failure
Left ventricular failure	I501	Heart Failure
Heart failure, unspecified	I509	Heart Failure
Acute myocardial infarction	I21	MI
Acute transmural myocardial infarction of anterior wall	I210	MI
Acute transmural myocardial infarction of inferior wall	I211	MI
Acute transmural myocardial infarction of other sites	I212	MI
Acute transmural myocardial infarction of unspecified site	I213	MI
Acute subendocardial myocardial infarction	I214	MI
Acute myocardial infarction, unspecified	I219	MI
Subsequent myocardial infarction	I22	MI
Subsequent myocardial infarction of anterior wall	I220	MI
Subsequent myocardial infarction of inferior wall	I221	MI
Subsequent myocardial infarction of other sites	I228	MI
Subsequent myocardial infarction of unspecified site	I229	MI
Certain current complications following acute myocardial infarction	I23	MI
Haemopericardium as current complication following acute myocardial infarction	I230	MI
Atrial septal defect as current complication following acute myocardial infarction	I231	MI
Ventricular septal defect as current complication following acute myocardial infarction	I232	MI
Rupture of cardiac wall without haemopericardium as current complication following acute myocardial infarction	I233	MI
Rupture of chordae tendineae as current complication following acute myocardial infarction	I234	MI
Rupture of papillary muscle as current complication following acute myocardial infarction	I235	MI
Thrombosis of atrium, auricular appendage, and ventricle as current complications following acute myocardial infarction	I236	MI
Other current complications following acute myocardial infarction	I238	MI
Dressler's syndrome	I241	MI
Old myocardial infarction	I252	MI

Abdominal aortic aneurysm, ruptured	I713	PAD AAA
Abdominal aortic aneurysm, without mention of rupture	I714	PAD AAA
Thoracoabdominal aortic aneurysm, ruptured	I715	PAD AAA
Thoracoabdominal aortic aneurysm, without mention of rupture	I716	PAD AAA
Aortic aneurysm of unspecified site, ruptured	I718	PAD AAA
Aortic aneurysm of unspecified site, without mention of rupture	I719	PAD AAA
Thromboangiitis obliterans [Buerger]	I731	PAD AAA
Other specified peripheral vascular diseases	I738	PAD AAA
Peripheral vascular disease, unspecified	I739	PAD AAA
Embolism and thrombosis of arteries of lower extremities	I743	PAD AAA
Embolism and thrombosis of arteries of extremities, unspecified	I744	PAD AAA
Embolism and thrombosis of iliac artery	I745	PAD AAA
Other transient cerebral ischaemic attacks and related syndromes	G458	Stroke
Transient cerebral ischaemic attack, unspecified	G459	Stroke
Middle cerebral artery syndrome	G460	Stroke
Anterior cerebral artery syndrome	G461	Stroke
Posterior cerebral artery syndrome	G462	Stroke
Brain stem stroke syndrome	G463	Stroke
Cerebellar stroke syndrome	G464	Stroke
Pure motor lacunar syndrome	G465	Stroke
Pure sensory lacunar syndrome	G466	Stroke
Other lacunar syndromes	G467	Stroke
Subarachnoid haemorrhage	I60	Stroke
Subarachnoid haemorrhage from carotid siphon and bifurcation	I600	Stroke
Subarachnoid haemorrhage from middle cerebral artery	I601	Stroke
Subarachnoid haemorrhage from anterior communicating artery	I602	Stroke
Subarachnoid haemorrhage from posterior communicating artery	I603	Stroke
Subarachnoid haemorrhage from basilar artery	I604	Stroke
Subarachnoid haemorrhage from vertebral artery	I605	Stroke
Subarachnoid haemorrhage from other intracranial arteries	I606	Stroke
Subarachnoid haemorrhage from intracranial artery, unspecified	I607	Stroke
Other subarachnoid haemorrhage	I608	Stroke
Subarachnoid haemorrhage, unspecified	I609	Stroke
Intracerebral haemorrhage	I61	Stroke
Intracerebral haemorrhage in hemisphere, subcortical	I610	Stroke
Intracerebral haemorrhage in hemisphere, cortical	I611	Stroke
Intracerebral haemorrhage in hemisphere, unspecified	I612	Stroke
Intracerebral haemorrhage in brain stem	I613	Stroke
Intracerebral haemorrhage in cerebellum	I614	Stroke
Intracerebral haemorrhage, intraventricular	I615	Stroke
Intracerebral haemorrhage, multiple localized	I616	Stroke
Other intracerebral haemorrhage	I618	Stroke
Intracerebral haemorrhage, unspecified	I618	Stroke
Nontraumatic intracerebral hemorrhage, unspecified	I619	Stroke
Other nontraumatic intracranial haemorrhage	I62	Stroke
Subdural haemorrhage (acute)(nontraumatic)	I620	Stroke
Nontraumatic extradural haemorrhage	I621	Stroke
Intracranial haemorrhage (nontraumatic), unspecified	I629	Stroke

Cerebral infarction	I63	Stroke
Cerebral infarction due to thrombosis of precerebral arteries	I630	Stroke
Cerebral infarction due to embolism of precerebral arteries	I631	Stroke
Cerebral infarction due to unspecified occlusion or stenosis of precerebral arteries	I632	Stroke
Cerebral infarction due to thrombosis of cerebral arteries	I633	Stroke
Cerebral infarction due to embolism of cerebral arteries	I634	Stroke
Cerebral infarction due to unspecified occlusion or stenosis of cerebral arteries	I635	Stroke
Cerebral infarction due to cerebral venous thrombosis, nonpyogenic	I636	Stroke
Other cerebral infarction	I638	Stroke
Cerebral infarction, unspecified	I639	Stroke
Stroke, not specified as haemorrhage or infarction	I64	Stroke
Sequelae of subarachnoid haemorrhage	I690	Stroke
Sequelae of intracerebral haemorrhage	I691	Stroke
Sequelae of other nontraumatic intracranial haemorrhage	I692	Stroke
Sequelae of cerebral infarction	I693	Stroke
Sequelae of stroke, not specified as haemorrhage or infarction	I694	Stroke
Sequelae of other and unspecified cerebrovascular diseases	I698	Stroke
Cardiac arrest	I46	VA Cardiac arrest/death
Cardiac arrest with successful resuscitation	I460	VA Cardiac arrest/death
Sudden cardiac death, so described	I461	VA Cardiac arrest/death
Cardiac arrest, unspecified	I469	VA Cardiac arrest/death
Re-entry ventricular arrhythmia	I470	VA Cardiac arrest/death
Ventricular fibrillation and flutter	I490	VA Cardiac arrest/death

**TERM**	**OPCS code**	**CATEGORY**
Saphenous vein graft replacement of coronary artery	K40	CABG
Saphenous vein graft replacement of one coronary artery	K40.1	CABG
Saphenous vein graft replacement of two coronary arteries	K40.2	CABG
Saphenous vein graft replacement of three coronary arteries	K40.3	CABG
Saphenous vein graft replacement of four or more coronary arteries	K40.4	CABG
Other specified saphenous vein graft replacement of coronary artery	K40.8	CABG
Unspecified saphenous vein graft replacement of coronary artery	K40.9	CABG
Other autograft replacement of coronary artery	K41	CABG
Autograft replacement of one coronary artery NEC	K41.1	CABG
Autograft replacement of two coronary arteries NEC	K41.2	CABG
Autograft replacement of three coronary arteries NEC	K41.3	CABG
Autograft replacement of four or more coronary arteries NEC	K41.4	CABG
Other specified other autograft replacement of coronary artery	K41.8	CABG
Unspecified other autograft replacement of coronary artery	K41.9	CABG
Allograft replacement of coronary artery	K42	CABG
Allograft replacement of one coronary artery	K42.1	CABG
Allograft replacement of two coronary arteries	K42.2	CABG
Allograft replacement of three coronary arteries	K42.3	CABG
Allograft replacement of four or more coronary arteries	K42.4	CABG

Other specified allograft replacement of coronary artery	K42.8	CABG
Unspecified allograft replacement of coronary artery	K42.9	CABG
Prosthetic replacement of coronary artery	K43	CABG
Prosthetic replacement of one coronary artery	K43.1	CABG
Prosthetic replacement of two coronary arteries	K43.2	CABG
Prosthetic replacement of three coronary arteries	K43.3	CABG
Prosthetic replacement of four or more coronary arteries	K43.4	CABG
Other specified prosthetic replacement of coronary artery	K43.8	CABG
Unspecified prosthetic replacement of coronary artery	K43.9	CABG
Other replacement of coronary artery	K44	CABG
Replacement of coronary arteries using multiple methods	K44.1	CABG
Revision of replacement of coronary artery	K44.2	CABG
Other specified other replacement of coronary artery	K44.8	CABG
Unspecified other replacement of coronary artery	K44.9	CABG
Connection of thoracic artery to coronary artery	K45	CABG
Double anastomosis of mammary arteries to coronary arteries	K45.1	CABG
Double anastomosis of thoracic arteries to coronary arteries NEC	K45.2	CABG
Anastomosis of mammary artery to left anterior descending coronary artery	K45.3	CABG
Anastomosis of mammary artery to coronary artery NEC	K45.4	CABG
Anastomosis of thoracic artery to coronary artery NEC	K45.5	CABG
Revision of connection of thoracic artery to coronary artery	K45.6	CABG
Other specified connection of thoracic artery to coronary artery	K45.8	CABG
Unspecified connection of thoracic artery to coronary artery	K45.9	CABG
Other bypass of coronary artery	K46	CABG
Double implantation of mammary arteries into heart	K46.1	CABG
Double implantation of thoracic arteries into heart NEC	K46.2	CABG
Implantation of mammary artery into heart NEC	K46.3	CABG
Implantation of thoracic artery into heart NEC	K46.4	CABG
Revision of implantation of thoracic artery into heart	K46.5	CABG
Other specified other bypass of coronary artery	K46.8	CABG
Unspecified other bypass of coronary artery	K46.9	CABG
Emergency replacement of aneurysmal segment of aorta	L18	PAD AAA
Emergency replacement of aneurysmal segment of ascending aorta by anastomosis of aorta to aorta	L181	PAD AAA
Emergency replacement of aneurysmal segment of thoracic aorta by anastomosis of aorta to aorta NEC	L182	PAD AAA
Emergency replacement of aneurysmal segment of suprarenal abdominal aorta by anastomosis of aorta to aorta	L183	PAD AAA
Emergency replacement of aneurysmal segment of infrarenal abdominal aorta by anastomosis of aorta to aorta	L184	PAD AAA
Emergency replacement of aneurysmal segment of abdominal aorta by anastomosis of aorta to aorta NEC	L185	PAD AAA
Emergency replacement of aneurysmal bifurcation of aorta by anastomosis of aorta to iliac artery	L186	PAD AAA
Other specified emergency replacement of aneurysmal segment of aorta	L188	PAD AAA
Unspecified emergency replacement of aneurysmal segment of aorta	L189	PAD AAA

Other replacement of aneurysmal segment of aorta	L19	PAD AAA
Replacement of aneurysmal segment of ascending aorta by anastomosis of aorta to aorta NEC	L191	PAD AAA
Replacement of aneurysmal segment of thoracic aorta by anastomosis of aorta to aorta NEC	L192	PAD AAA
Replacement of aneurysmal segment of suprarenal abdominal aorta by anastomosis of aorta to aorta NEC	L193	PAD AAA
Replacement of aneurysmal segment of infrarenal abdominal aorta by anastomosis of aorta to aorta NEC	L194	PAD AAA
Replacement of aneurysmal segment of abdominal aorta by anastomosis of aorta to aorta NEC	L195	PAD AAA
Replacement of aneurysmal bifurcation of aorta by anastomosis of aorta to iliac artery NEC	L196	PAD AAA
Other specified other replacement of aneurysmal segment of aorta	L198	PAD AAA
Unspecified other replacement of aneurysmal segment of aorta	L199	PAD AAA
Other emergency bypass of segment of aorta	L20	PAD AAA
Emergency bypass of segment of ascending aorta by anastomosis of aorta to aorta NEC	L201	PAD AAA
Emergency bypass of segment of thoracic aorta by anastomosis of aorta to aorta NEC	L202	PAD AAA
Emergency bypass of segment of suprarenal abdominal aorta by anastomosis of aorta to aorta NEC	L203	PAD AAA
Emergency bypass of segment of infrarenal abdominal aorta by anastomosis of aorta to aorta NEC	L204	PAD AAA
Emergency bypass of segment of abdominal aorta by anastomosis of aorta to aorta NEC	L205	PAD AAA
Emergency bypass of bifurcation of aorta by anastomosis of aorta to iliac artery NEC	L206	PAD AAA
Other specified other emergency bypass of segment of aorta	L208	PAD AAA
Unspecified other emergency bypass of segment of aorta	L209	PAD AAA
Operations on aneurysm of aorta NEC	L254	PAD AAA
Transluminal insertion of stent graft for aneurysmal segment of aorta	L27	PAD AAA
Endovascular insertion of stent graft for infrarenal abdominal aortic aneurysm	L271	PAD AAA
Endovascular insertion of stent graft for suprarenal aortic aneurysm	L272	PAD AAA
Endovascular insertion of stent graft for thoracic aortic aneurysm	L273	PAD AAA
Endovascular insertion of stent graft for aortic dissection in any position	L274	PAD AAA
Endovascular insertion of stent graft for aortic aneurysm of bifurcation NEC	L275	PAD AAA
Endovascular insertion of stent graft for aorto-uniiliac aneurysm	L276	PAD AAA
Other specified transluminal insertion of stent graft for aneurysmal segment of aorta	L278	PAD AAA
Unspecified transluminal insertion of stent graft for aneurysmal segment of aorta	L279	PAD AAA
Transluminal operations on aneurysmal segment of aorta	L28	PAD AAA
Endovascular insertion of stent for infrarenal abdominal aortic aneurysm	L281	PAD AAA
Endovascular insertion of stent for suprarenal aortic aneurysm	L282	PAD AAA

Endovascular insertion of stent for thoracic aortic aneurysm	L283	PAD AAA
Endovascular insertion of stent for aortic dissection in any position	L284	PAD AAA
Endovascular insertion of stent for aortic aneurysm of bifurcation NEC	L285	PAD AAA
Endovascular insertion of stent for aorto-uniiliac aneurysm	L286	PAD AAA
Other specified transluminal operations on aneurysmal segment of aorta	L288	PAD AAA
Unspecified transluminal operations on aneurysmal segment of aort	L289	PAD AAA
Other emergency bypass of iliac artery	L50	PAD AAA
Emergency bypass of common iliac artery by anastomosis of aorta to common iliac artery NEC	L50.1	PAD AAA
Emergency bypass of iliac artery by anastomosis of aorta to external iliac artery NEC	L50.2	PAD AAA
Emergency bypass of artery of leg by anastomosis of aorta to common femoral artery NEC	L50.3	PAD AAA
Emergency bypass of artery of leg by anastomosis of aorta to deep femoral artery NEC	L50.4	PAD AAA
Emergency bypass of iliac artery by anastomosis of iliac artery to iliac artery NEC	L50.5	PAD AAA
Emergency bypass of artery of leg by anastomosis of iliac artery to femoral artery NEC	L50.6	PAD AAA
Other specified other emergency bypass of iliac artery	L50.8	PAD AAA
Unspecified other emergency bypass of iliac artery	L50.9	PAD AAA
Other bypass of iliac artery	L51	PAD AAA
Bypass of common iliac artery by anastomosis of aorta to common iliac artery NEC	L51.1	PAD AAA
Bypass of iliac artery by anastomosis of aorta to external iliac artery NEC	L51.2	PAD AAA
Bypass of artery of leg by anastomosis of aorta to common femoral artery NEC	L51.3	PAD AAA
Bypass of artery of leg by anastomosis of aorta to deep femoral artery NEC	L51.4	PAD AAA
Bypass of iliac artery by anastomosis of iliac artery to iliac artery NEC	L51.5	PAD AAA
Bypass of artery of leg by anastomosis of iliac artery to femoral artery NEC	L51.6	PAD AAA
Other specified other bypass of iliac artery	L51.8	PAD AAA
Unspecified other bypass of iliac artery	L51.9	PAD AAA
Reconstruction of iliac artery	L52	PAD AAA
Endarterectomy of iliac artery and patch repair of iliac artery	L52.1	PAD AAA
Endarterectomy of iliac artery NEC	L52.2	PAD AAA
Other specified reconstruction of iliac artery	L52.8	PAD AAA
Unspecified reconstruction of iliac artery	L52.9	PAD AAA
Other open operations on iliac artery	L53	PAD AAA
Repair of iliac artery NEC	L53.1	PAD AAA
Open embolectomy of iliac artery	L53.2	PAD AAA
Percutaneous transluminal angioplasty of iliac artery	L54.1	PAD AAA
Percutaneous transluminal embolectomy of iliac artery	L54.2	PAD AAA
Percutaneous transluminal insertion of stent into iliac artery	L54.4	PAD AAA
Other specified transluminal operations on iliac artery	L54.8	PAD AAA

Unspecified transluminal operations on iliac artery	L54.9	PAD AAA
Other emergency bypass of femoral artery	L58	PAD AAA
Emergency bypass of femoral artery by anastomosis of femoral artery to femoral artery NEC	L58.1	PAD AAA
Emergency bypass of femoral artery by anastomosis of femoral artery to popliteal artery using prosthesis NEC	L58.2	PAD AAA
Emergency bypass of femoral artery by anastomosis of femoral artery to popliteal artery using vein graft NEC	L58.3	PAD AAA
Emergency bypass of femoral artery by anastomosis of femoral artery to tibial artery using prosthesis NEC	L58.4	PAD AAA
Emergency bypass of femoral artery by anastomosis of femoral artery to tibial artery using vein graft NEC	L58.5	PAD AAA
Emergency bypass of femoral artery by anastomosis of femoral artery to peroneal artery using prosthesis NEC	L58.6	PAD AAA
Emergency bypass of femoral artery by anastomosis of femoral artery to peroneal artery using vein graft NEC	L58.7	PAD AAA
Other specified other emergency bypass of femoral artery	L58.8	PAD AAA
Unspecified other emergency bypass of femoral artery	L58.9	PAD AAA
Other bypass of femoral artery	L59	PAD AAA
Bypass of femoral artery by anastomosis of femoral artery to femoral artery NEC	L59.1	PAD AAA
Bypass of femoral artery by anastomosis of femoral artery to popliteal artery using prosthesis NEC	L59.2	PAD AAA
Bypass of femoral artery by anastomosis of femoral artery to popliteal artery using vein graft NEC	L59.3	PAD AAA
Bypass of femoral artery by anastomosis of femoral artery to tibial artery using prosthesis NEC	L59.4	PAD AAA
Bypass of femoral artery by anastomosis of femoral artery to tibial artery using vein graft NEC	L59.5	PAD AAA
Bypass of femoral artery by anastomosis of femoral artery to peroneal artery using prosthesis NEC	L59.6	PAD AAA
Bypass of femoral artery by anastomosis of femoral artery to peroneal artery using vein graft NEC	L59.7	PAD AAA
Other specified other bypass of femoral artery	L59.8	PAD AAA
Unspecified other bypass of femoral artery	L59.9	PAD AAA
Reconstruction of femoral artery	L60	PAD AAA
Endarterectomy of femoral artery and patch repair of femoral artery	L60.1	PAD AAA
Endarterectomy of femoral artery NEC	L60.2	PAD AAA
Profundoplasty of femoral artery and patch repair of deep femoral artery	L60.3	PAD AAA
Profundoplasty of femoral artery NEC	L60.4	PAD AAA
Other specified reconstruction of femoral artery	L60.8	PAD AAA
Unspecified reconstruction of femoral artery	L60.9	PAD AAA
Other open operations on femoral artery	L62	PAD AAA
Repair of femoral artery NEC	L62.1	PAD AAA
Open embolectomy of femoral artery	L62.2	PAD AAA
Other specified other open operations on femoral artery	L62.8	PAD AAA
Unspecified other open operations on femoral artery	L62.9	PAD AAA
Percutaneous transluminal angioplasty of femoral artery	L63.1	PAD AAA
Percutaneous transluminal embolectomy of femoral artery	L63.2	PAD AAA
Percutaneous transluminal embolisation of femoral artery	L63.3	PAD AAA

Percutaneous transluminal insertion of stent into femoral artery	L63.5	PAD AAA
Revision of reconstruction of artery	L65	PAD AAA
Revision of reconstruction involving aorta	L65.1	PAD AAA
Revision of reconstruction involving iliac artery	L65.2	PAD AAA
Revision of reconstruction involving femoral artery	L65.3	PAD AAA
Transluminal balloon angioplasty of coronary artery	K49	PCI
Percutaneous transluminal balloon angioplasty of one coronary artery	K49.1	PCI
Percutaneous transluminal balloon angioplasty of multiple coronary arteries	K49.2	PCI
Percutaneous transluminal balloon angioplasty of bypass graft of coronary artery	K49.3	PCI
Percutaneous transluminal cutting balloon angioplasty of coronary artery	K49.4	PCI
Other specified transluminal balloon angioplasty of coronary artery	K49.8	PCI
Unspecified transluminal balloon angioplasty of coronary artery	K49.9	PCI
Other therapeutic transluminal operations on coronary artery	K50	PCI
Percutaneous transluminal laser coronary angioplasty	K50.1	PCI
Percutaneous transluminal atherectomy of coronary artery	K50.4	PCI
Other specified other therapeutic transluminal operations on coronary artery	K50.8	PCI
Unspecified other therapeutic transluminal operations on coronary artery	K50.9	PCI
Percutaneous transluminal balloon angioplasty and insertion of stent into coronary artery	K75	PCI
Percutaneous transluminal balloon angioplasty and insertion of 1-2 drug-eluting stents into coronary artery	K75.1	PCI
Percutaneous transluminal balloon angioplasty and insertion of 3 or more drug-eluting stents into coronary artery	K75.2	PCI
Percutaneous transluminal balloon angioplasty and insertion of 1-2 stents into coronary artery	K75.3	PCI
Percutaneous transluminal balloon angioplasty and insertion of 3 or more stents into coronary artery NEC	K75.4	PCI
Other specified percutaneous transluminal balloon angioplasty and insertion of stent into coronary artery	K75.8	PCI
Unspecified percutaneous transluminal balloon angioplasty and insertion of stent into coronary artery	K75.9	PCI
External resuscitation	X50	VA Cardiac arrest/death
Advanced cardiac pulmonary resuscitation	X503	VA Cardiac arrest/death
External ventricular defibrillation	X504	VA Cardiac arrest/death
Other specified external resuscitation	X508	VA Cardiac arrest/death
Unspecified external resuscitation	X509	VA Cardiac arrest/death

Table S2 - Patient characteristics by type of chest pain in GOLD, *n* (%) unless stated

		Chest pain Non-coronary	Chest pain Unattributed	Angina
n		89,145	226,186	10,047
Age: Mean (SD)		45.6 (16.93)	47.3 (16.60)	66.0 (12.22)
Female		52,146 (58)	120,046 (53)	4,909 (49)
Ethnicity:	White	74,726 (93)	188,651 (92)	9,326 (96)
Deprivation:	Least	21,683 (24)	55,028 (24)	2,353 (23)
	2nd	19,541 (22)	51,713 (23)	2,212 (22)
	3rd	18,215 (20)	46,337 (21)	2,066 (21)
	4th	17,720 (20)	43,484 (19)	2,071 (21)
	Most	11,920 (13)	29,464 (13)	1,333 (13)
Risk factors				
Smoking:	Current	27,822 (32)	65,926 (30)	2,134 (22)
	Ex	16,916 (20)	46,757 (21)	3,517 (36)
	Never	41,646 (48)	107,471 (49)	4,176 (42)
Diabetes:	Type 1	153 (<1)	372 (<1)	35 (<1)
	Type 2	3,080 (3)	8,885 (4)	1,385 (14)
FH: angina/heart attack <60yrs		1,663 (2)	6,439 (3)	560 (6)
Chronic Kidney Disease stage 3-5		2,744 (3)	8,140 (4)	1,044 (10)
Atrial fibrillation		448 (<1)	2,225 (1)	437 (4)
Treated hypertension		14,120 (16)	45,122 (20)	8,441 (84)
Migraine		2,594 (3)	6,347 (3)	140 (1)
Rheumatoid arthritis		442 (<1)	1,031 (<1)	81 (<1)
Severe mental illness		1,799 (2)	4,530 (2)	176 (2)
Corticosteroid medication		4,429 (5)	11,437 (5)	879 (9)
BMI: Mean (SD)		26.2 (5.65)	26.7 (5.67)	28.3 (5.55)
Cholesterol/HDL ratio: Mean (SD)		3.8 (1.26)	3.9 (1.28)	3.8 (1.30)
Alternative explanation /comorbidity				
Depression/anxiety		13,092 (15)	33,401 (15)	1,199 (12)
Oesophageal reflux		7,310 (8)	22,246 (10)	1,313 (13)
Respiratory		18,487 (21)	46,736 (21)	2,547 (25)
Osteoarthritis		3,139 (4)	8,150 (4)	880 (9)

Low back pain	17,704 (20)	42,617 (19)	1,973 (20)
Neck pain	7,327 (8)	17,510 (8)	840 (8)
Cancer	1,756 (2)	4,265 (2)	285 (3)
QRISK3: Median (IQR)	2.38 (0.49, 8.74)	3.37 (0.77, 10.79)	N/A

FH: family history; IQR: interquartile range; SD: standard deviation. Complete data range: Ethnicity 90-97%; smoking 97-98%; BMI 85-92%, Cholesterol/HDL ratio 36-77%

Table S3 –Associations of cardiovascular events with unattributed chest pain (compared to non-coronary chest pain) by year of index presentation in Aurum (maximum 3-year follow-up)

	Year of index presentation			
	2002	2006	2010	2014
	HR* (95% CI)	HR* (95% CI)	HR* (95% CI)	HR* (95% CI)
Any cardiovascular	1.29 (1.22, 1.36)	1.28 (1.24, 1.32)	1.27 (1.23, 1.30)	1.26 (1.20, 1.31)
Coronary	1.48 (1.38, 1.58)	1.48 (1.42, 1.55)	1.49 (1.43, 1.55)	1.50 (1.41, 1.58)
Myocardial infarction	1.11 (0.96, 1.29)	1.16 (1.05, 1.27)	1.20 (1.11, 1.29)	1.25 (1.12, 1.39)
Stroke	1.03 (0.91, 1.16)	1.00 (0.93, 1.08)	0.98 (0.93, 1.04)	0.96 (0.88, 1.04)

* adjusted for age, gender, ethnicity, neighbourhood deprivation, year of index presentation, smoking status, type 1 diabetes, type 2 diabetes, family history of coronary heart disease, chronic kidney disease, atrial fibrillation, treated hypertension, migraine, rheumatoid arthritis, severe mental illness, corticosteroid medication, BMI, depression/anxiety, oesophageal reflux, respiratory, osteoarthritis, lower back pain, neck pain, cancer. HR: hazard ratio; CI: confidence interval

Table S4 –Associations of cardiovascular events with unattributed chest pain (compared to non-coronary chest pain) at different points during follow-up in GOLD

	Time since index date			
	12 months<br>HR* (95% CI)	36 months<br>HR* (95% CI)	60 months<br>HR* (95% CI)	120 months<br>HR* (95% CI)
Any cardiovascular	1.23 (1.18, 1.29)	1.06 (1.02, 1.11)	1.04 (1.01, 1.08)	1.04 (0.97, 1.10)
Coronary	1.45 (1.37, 1.54)	1.18 (1.11, 1.24)	1.11 (1.06, 1.17)	1.03 (0.96, 1.11)
Myocardial infarction	1.17 (1.03, 1.33)	1.17 (1.05, 1.31)	1.12 (1.01, 1.23)	0.98 (0.87, 1.12)
Stroke	1.04 (0.94, 1.14)	0.99 (0.90, 1.08)	0.99 (0.92, 1.07)	1.04 (0.93, 1.17)

* fully adjusted model; non-coronary group is the reference group. HR: hazard ratio; CI: confidence interval

Table S5 – Incidence of types of cardiovascular events and associations with unattributed chest pain in GOLD

	Number at risk	Number (%) with event	Rate per 10,000 person-years	Unadjusted HR (95% CI)	Model 1 Adjusted* HR (95% CI)	Model 2 Adjusted† HR (95% CI)	Model 3 Adjusted‡ HR (95% CI)
Coronary							
Non-coronary	89,145	4,083 (4.6)	86.74 (84.12, 89.44)	1.00	1.00	1.00	1.00
Unattributed	226,186	14,659 (6.5)	123.54 (121.56, 125.56)	1.42 (1.38, 1.47)	1.28 (1.24, 1.33)	1.24 (1.20, 1.29)	1.24 (1.20, 1.28)
Myocardial infarction							
Non-coronary	89,145	1,131 (1.3)	23.47 (22.14, 24.88)	1.00	1.00	1.00	1.00
Unattributed	226,186	3,697 (1.6)	29.99 (29.04, 30.97)	1.28 (1.19, 1.37)	1.12 (1.04, 1.20)	1.11 (1.03, 1.19)	1.11 (1.03, 1.19)
Angina	10,047	958 (9.5)	168.90 (158.54, 179.95)	7.14 (6.49, 7.85)	2.55 (2.32, 2.80)	2.15 (1.95, 2.38)	2.17 (1.97, 2.39)
Stroke							
Non-coronary	89,145	1,766 (2.0)	36.80 (35.13, 38.56)	1.00	1.00	1.00	1.00
Unattributed	226,186	5,118 (2.3)	41.68 (40.55, 42.84)	1.13 (1.07, 1.20)	1.02 (0.96, 1.08)	1.00 (0.95, 1.07)	1.01 (0.95, 1.07)
Angina	9,987	696 (7.0)	122.06 (113.32, 131.48)	3.29 (2.99, 3.63)	1.14 (1.04, 1.26)	1.02 (0.92, 1.13)	1.03 (0.93, 1.14)

* adjusted for age, gender, ethnicity, neighbourhood deprivation, year of index presentation; † additionally adjusted for smoking status, type 1 diabetes, type 2 diabetes, family history of coronary heart disease, chronic kidney disease, atrial fibrillation, treated hypertension, migraine, rheumatoid arthritis, severe mental illness, corticosteroid medication, BMI; ‡ additionally adjusted for depression/anxiety, oesophageal reflux, respiratory, osteoarthritis, lower back pain, neck pain, cancer. HR: hazard ratio; CI: confidence interval

Figure S1 – Change in risk of cardiovascular event over follow-up*

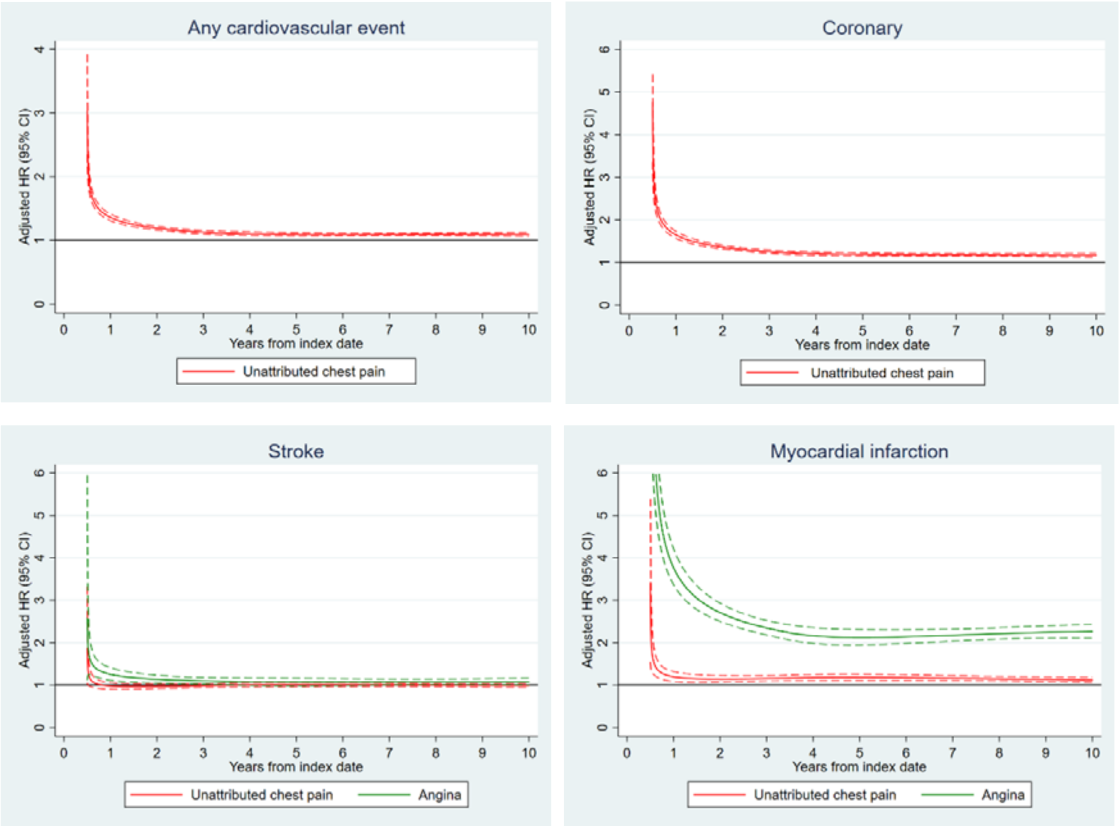

* Follow-up starts at 6 months after index date (i.e. end of diagnosis window); reference group is non-coronary chest pain
